# The lipid flippase SLC47A1 blocks metabolic vulnerability to ferroptosis

**DOI:** 10.1038/s41467-022-35707-2

**Published:** 2022-12-27

**Authors:** Zhi Lin, Jiao Liu, Fei Long, Rui Kang, Guido Kroemer, Daolin Tang, Minghua Yang

**Affiliations:** 1 Department of Pediatrics, The Third Xiangya Hospital, Central South University, Changsha, Hunan 410013 China; 2 DAMP Laboratory, Third Affiliated Hospital of Guangzhou Medical University, Guangzhou, Guangdong 510150 China; 3 Guangzhou Municipal and Guangdong Provincial Key Laboratory of Protein Modification and Degradation, Guangzhou Medical University, Guangzhou, Guangdong 511436 China; 4 Department of Gastrointestinal Surgery, The Third Xiangya Hospital, Central South University, Changsha, Hunan 410013 China; 5 Department of Surgery, UT Southwestern Medical Center, Dallas, Texas 75390 USA; 6 Centre de Recherche des Cordeliers, Equipe labellisée par la Ligue contre le cancer, Université de Paris, Sorbonne Université, INSERM U1138, Institut Universitaire de France, Paris, France; 7 Metabolomics and Cell Biology Platforms, Gustave Roussy Cancer Campus, Villejuif, France; 8 Pôle de Biologie, Hôpital Européen Georges Pompidou, AP-HP, Paris, France

**Keywords:** Cell death, Cell signalling, Membrane lipids

## Abstract

Ferroptosis is a type of regulated necrosis caused by unrestricted lipid peroxidation and subsequent plasma membrane rupture. However, the lipid remodeling mechanism that determines sensitivity to ferroptosis remains poorly understood. Here, we report a previously unrecognized role for the lipid flippase solute carrier family 47 member 1 (SLC47A1) as a regulator of lipid remodeling and survival during ferroptosis. Among 49 phospholipid scramblases, flippases, and floppases we analyzed, only SLC47A1 had mRNA that was selectively upregulated in multiple cancer cells exposed to ferroptotic inducers. Large-scale lipidomics and functional analyses revealed that the silencing of SLC47A1 increased RSL3- or erastin-induced ferroptosis by favoring ACSL4-SOAT1–mediated production of polyunsaturated fatty acid cholesterol esters. We identified peroxisome proliferator activated receptor alpha (PPARA) as a transcription factor that transactivates SLC47A1. The depletion of PPARA and SLC47A1 similarly sensitized cells to ferroptosis induction, whereas transfection-enforced re-expression of SLC47A1 restored resistance to ferroptosis in PPARA-deficient cells. Pharmacological or genetic blockade of the PPARA-SLC47A1 pathway increased the anticancer activity of a ferroptosis inducer in mice. These findings establish a direct molecular link between ferroptosis and lipid transporters, which may provide metabolic targets for overcoming drug resistance.

## Abstract (web summary)

Ferroptosis is cell death caused by lipid peroxidation and plasma membrane rupture. Here, the authors report an additional role for the lipid flippase SLC47A1 as a regulator of lipid remodeling and survival during ferroptosis, thereby providing a metabolic target for overcoming drug resistance.

## Introduction

Ferroptosis is a type of iron-dependent regulated cell death driven by unrestricted lipid peroxidation in the plasma membrane or membrane organelles^1–3^. Dysregulated ferroptosis is implicated in pathological conditions and is becoming an emerging therapeutic target for the treatment of cancer^4^. The classic ferroptosis inducers erastin and RSL3 were initially identified by screening small molecule compounds able to suppress oncogenic *RAS* mutation-driven tumor growth and were later found to block the antioxidant system relying on xc^−^-glutathione peroxidase 4 (GPX4)^5,6^. However, cancer cells can activate or remodel stress pathways to avoid cell death, which limits the activity of ferroptosis activators^4^. Although the sensitivity to ferroptosis is tightly linked to various biochemical pathways, lipid metabolism is fundamental for shaping ferroptotic response^7^. For example, acyl-CoA synthetase long-chain family member 4 (ACSL4) drives ferroptosis through the oxidization of polyunsaturated fatty acids (PUFAs)^8–10^, while peroxisomes favor lipid peroxidation through the biosynthesis of plasmalogens^11^. Lipophagy, the autophagic degradation of intracellular lipid droplets, provides an additional lipid resource for oxidative damage and ferroptosis^12^. Thus, a detailed understanding of the impact of lipid metabolism on ferroptosis and its avoidance may yield insights that allow us to improve ferroptosis induction in cancer cells^7^.

Asymmetric distribution of lipids across the two leaflets of biological membranes is a general feature of eukaryotic cells^13^. Scramblases, flippases, and floppases are the main types of membrane transporters that facilitate lipid translocation across the plasma membrane, thereby affecting intracellular lipid homeostasis^14^. Abnormal expression and activation of phospholipid transporters are associated with cell death and pathological conditions including malignancy^15,16^. Although the appearance of anionic phospholipid phosphatidylserine on the exoplasmic face is a pathognomonic and well-studied feature of apoptosis^17^, the function of phospholipid transporters in ferroptosis has not been studied in detail.

In this study, we systematically measured the expression of genes encoding phospholipid transporters and identified solute carrier family 47 member 1 (SLC47A1) as the most significantly ferroptosis-induced gene. Metabolomics and functional analyses demonstrated that peroxisome proliferator-activated receptor alpha (PPARA)-dependent SLC47A1 expression blocks ferroptosis by inhibiting the production of PUFA esterified cholesterol esters (CEs) for lipid peroxidation. Animal experiments confirmed that targeting the PPARA-SLC47A1 pathway enhances ferroptosis-mediated tumor suppression. These results not only reveal a new metabolic checkpoint of ferroptosis resistance, but also establish an approach to improve the antineoplastic activity of ferroptosis inducers.

## Results

### SLC47A1 is upregulated during ferroptosis

**Fig. 1:**
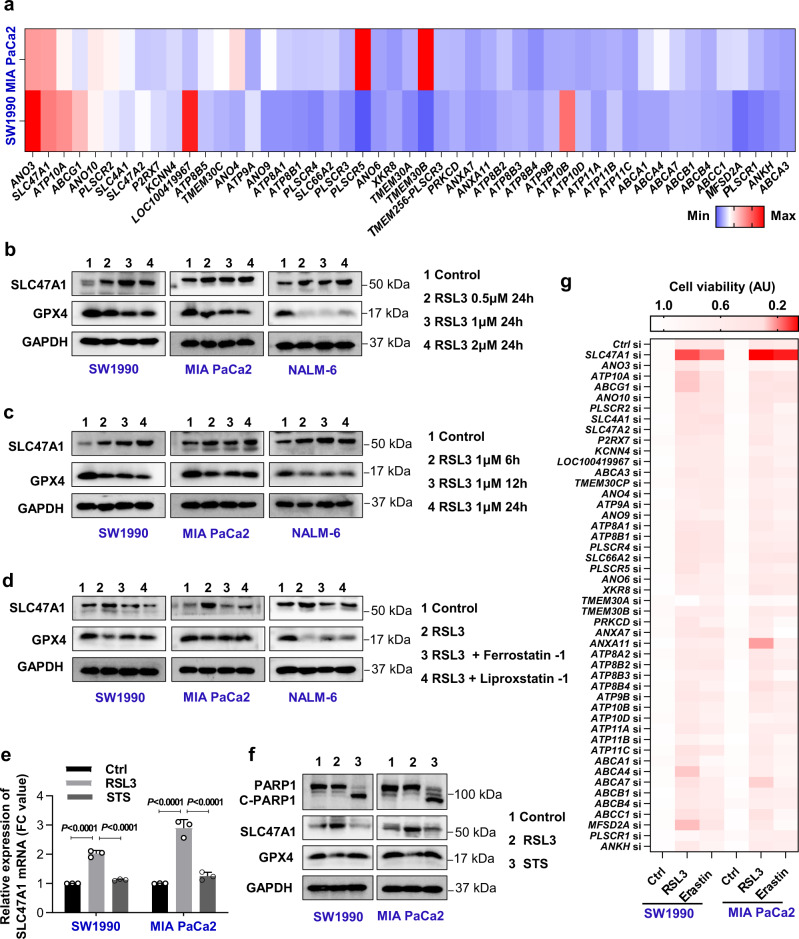
SLC47A1 is upregulated during ferroptosis. **a** Relative mRNA change of phospholipid transporters in indicated human PDAC cells following treatment with RSL3 (1 μM) for 24 h. **b** Western blot analysis of protein expression in indicated cells following treatment with RSL3 (0.5–2 μM) for 24 h. **c** Western blot analysis of protein expression in indicated cells following treatment with RSL3 (1 μM) for 6–24 h. **d** Western blot analysis of protein expression in indicated cells following treatment with RSL3 (1 μM) in the absence or presence of liproxstatin-1 (0.5 μM) or ferrostatin-1 (0.5 μM) for 24 h. **e** qPCR analysis of *SLC47A1* mRNA expression in indicated cells following treatment with RSL3 (1 μM, 24 h) or staurosporine (1 μM, 12 h) (*n* = 3 biologically independent samples, statistical significance was analyzed using One-way ANOVA with Dunnett’s multiple comparisons test, data were presented as mean ± SD). **f** Western blot analysis of protein expression in indicated cells following treatment with RSL3 (1 μM, 24 h) or staurosporine (1 μM, 12 h). **g** Heatmap of relative levels of cell death in control and the indicated gene knockdown SW1990 and MIAPaCa2 cells following treatment with RSL3 (1 μM, 24 h) or erastin (10 mM, 24 h). **a**, **g** Data are shown as the mean of three biologically independent samples. **b**–**d**, **f** Each western blot data is representative of three independent experiments. Quantification results are provided in Supplementary Fig. 1. Exact *p* values provided as Source Data. Source data are provided as a Source Data file.

Human pancreatic ductal adenocarcinoma (PDAC) cells are widely used to investigate the molecular mechanisms of ferroptosis^18–24^. Compared with erastin, RSL3 has a greater capacity to induce ferroptosis by inhibiting GPX4 activity^5^ or causing its degradation^25,26^. To uncover the role of phospholipid transporters in ferroptosis, the mRNA levels of 49 genes that coded for phospholipid scramblases, flippases, and floppases were quantified in two human PDAC cell lines (SW1990 and MIA PaCa2). A qPCR analysis revealed that *SLC47A1* and anoctamin 3 (*ANO3*) ranked among the top two upregulated genes in SW1990 and MIA PaCa2 cells in response to RSL3 (Fig. 1a). Western blot analysis further confirmed that protein expression of SLC47A1 and ANO3 was dose- and time-dependently upregulated following RSL3 treatment. As a control, the protein expression of GPX4 was downregulated by RSL3 (Fig. 1b, c, and Supplementary Fig. 1a, b).

Next, we determined the effects of ferroptosis inhibitors, including liproxstatin-1 and ferrostatin-1, on the RSL3-induced upregulation of SLC47A1 and ANO3 protein expression. Both liproxstatin-1 and ferrostatin-1 partly inhibited the RSL3-induced upregulation of SLC47A1, but not that of ANO3 in PDAC cells. As a control, RSL3-induced GPX4 protein downregulation was inhibited by liproxstatin-1 and ferrostatin-1 (Fig. 1d and Supplementary Fig. 1c–e). RSL3-induced SLC47A1 upregulation and GPX4 downregulation were also observed in a human B cell leukemia cell line (NALM-6), and these effects were reversed by ferroptosis inhibitors (Fig. 1b–d and Supplementary Fig. 1a–c). In contrast, the mRNA and protein expression of SLC47A1 was not affected by the apoptosis activator staurosporine (Fig. 1e, f). As a positive control, staurosporine induced the apoptosis-associated cleavage of poly (ADP-Ribose) polymerase 1 (PARP1) (Fig. 1f), a caspase substrate^27^. Collectively, these findings indicate that ferroptosis but not apoptosis is associated with the upregulation of SLC47A1 at the mRNA and protein levels.

### SLC47A1 is a repressor of ferroptosis

**Fig. 2:**
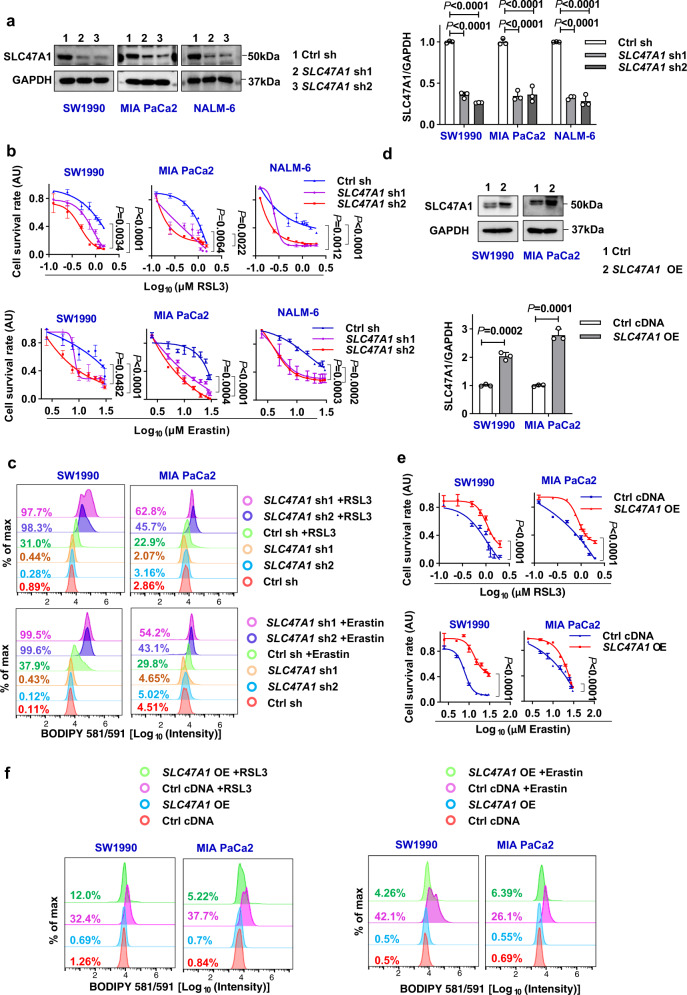
SLC47A1 is a repressor of ferroptosis. **a** Western blot analysis of protein expression in indicated control (Ctrl sh) and *SLC47A1*-knockdown (*SLC47A1* sh) PDAC cells. **b** Cell viability of control and *SLC47A1*-knockdown cells following treatment with RSL3 (1 μM) or erastin (10 μM) for 24 h. **c** Indicated cells were treated with RSL3 (1 μM) or erastin (10 μM) for 6 h, and then lipid ROS were assayed. **d** Western blot analysis of protein expression in indicated control (Ctrl cDNA) and *SLC47A1*-overexpresstion (*SLC47A1* OE) cells. **e** Cell viability of control and *SLC47A1*-overexpresstion cells following treatment with RSL3 or erastin for 24 h. **f** Indicated cells were treated with RSL3 (1 μM) or erastin (10 μM) for 6 h, and then lipid ROS were assayed. **a**, **d** Each western blot data is representative of three independent experiments, data were presented as mean ± SD, statistical significance was analyzed using one-way ANOVA with Dunnett’s multiple comparisons test, (**a**) or unpaired two-tailed T-test (**d**). **b**, **e** Data are shown with 3 biologically independent samples, data were presented as mean ± SD, and statistical significance was analyzed using two-way ANOVA with Dunnett’s multiple comparisons test. **c**, **f** The data is representative of three independent experiments. Quantification results are provided in Supplementary Fig. 2. Exact *p* values provided as Source Data. Source data are provided as a Source Data file.

To identify key phospholipid transporters involved in the regulation of ferroptosis, we constructed a siRNA library containing all phospholipid transporter genes (except 3 fusion genes) in SW1990 and MIA PaCa2 cells. This unbiased screen demonstrated the unique role of SLC47A1 in inhibiting erastin- and RSL3-induced ferroptosis in SW1990 and MIA PaCa2 cells, although ANXA11 may be selectively involved in inhibiting RSL3-induced ferroptosis in MIA PaCa2 cells, rather than SW1990 cells (Fig. 1g). Consistent with the siRNA screen, the knockdown of *SLC47A1* using small hairpin RNA (shRNA) also sensitized RSL3 and/or erastin to growth inhibition in PDAC (Fig. 2a, b), lung tumor cells (A549), breast tumor cells (MCF-7) and ovarian tumor cells (SKOV-3) (Supplementary Fig. 2a).

A morphological hallmark of ferroptosis is the rupture of the plasma membrane^28–30^. As detected by staining with the vital dye propidium iodide (PI), the knockdown of *SLC47A1* increased plasma membrane permeabilization and cell death induced by RSL3 or erastin (Supplementary Fig. 2b). Erastin- or RSL3-induced cell death in wild type and *SLC47A1*-knockdown cells was blocked by a ferroptosis inhibitor (ferrostatin-1), but not by an apoptosis inhibitor (ZVAD-FMK) or a necroptosis inhibitor (necrostatin-1) (Supplementary Fig. 2b**)**. Thus, the increased cell death of *SLC47A1*-knockdown cells is mediated by ferroptosis.

We next used BODIPY-C11 (a lipid-soluble ratiometric fluorescent indicator of lipid peroxidation) to estimate the level of ferroptosis-associated lipid peroxidation in wild type and *SLC47A1*-knockdown cells. The depletion of *SLC47A1* exacerbated RSL3- and erastin-induced lipid peroxidation (Fig. 2c and Supplementary Fig. 2c). Accordingly, *SLC47A1* knockdown also enhanced RSL3 and erastin-induced the accumulation of intracellular malondialdehyde (MDA) (Supplementary Fig. 2d), which is one of the final products of lipid peroxidation. In contrast, transfection-enforced overexpression of *SLC47A1* promoted the resistance of SW1990 and MIA PaCa2 cells to RSL3- or erastin-induced growth inhibition, cell death, and lipid peroxidation (Fig. 2d–f and Supplementary Fig. 2e, f). In sharp contrast, the knockdown or overexpression of *SLC47A1* failed to affect the pro-apoptotic activity of staurosporine on SW1990 and MIA PaCa2 cells (Supplementary Fig. 2g). Taken together, these data indicate that SLC47A1 acts as an endogenous repressor of ferroptosis.

### SLC47A1 regulates lipid remodeling in ferroptosis

**Fig. 3:**
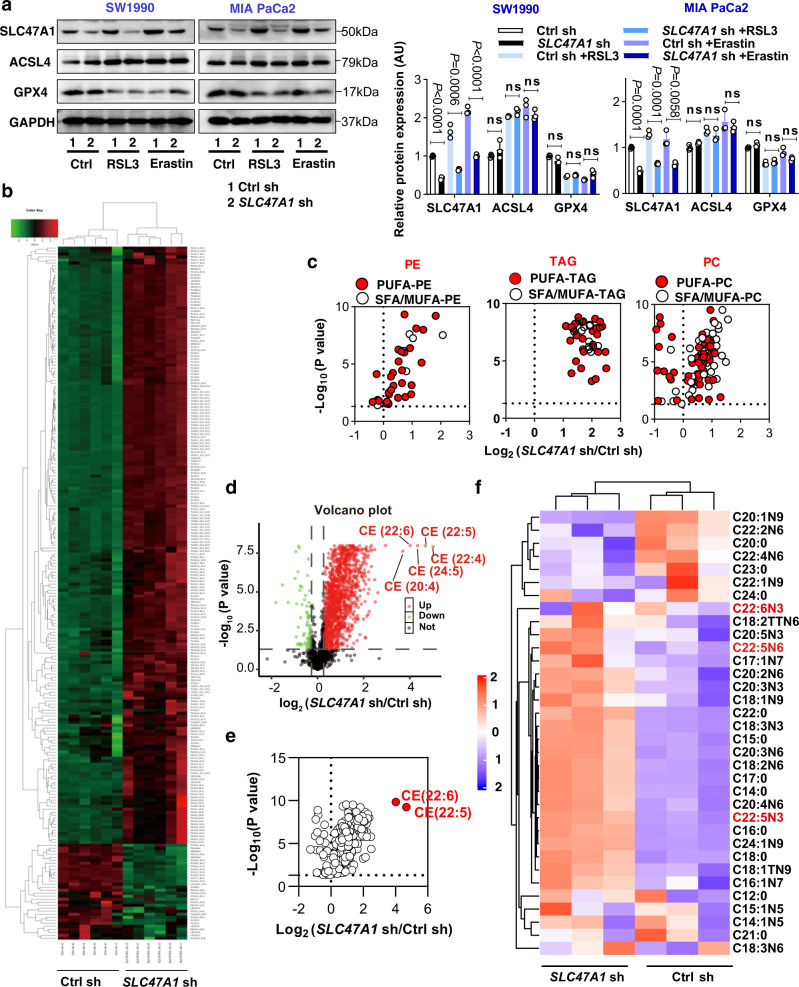
SLC47A1 regulates lipid remodeling in ferroptosis. **a** Western blot analysis of protein expression in indicated cells following treatment with RSL3 (1 μM) and erastin (10 μM) for 24 h. The data is representative of three independent experiments, data were presented as mean ± SD. Statistical significance was analyzed using two-way ANOVA with Dunnett’s multiple comparisons test. ns = not significant. Exact p values provided as Source Data. Source data are provided as a Source Data file. **b** Heatmap representing the relative lipid abundances in control and *SLC47A1*-knockdown SW1990 cells. Data are shown with six biologically independent samples. The relative abundance of lipid is color-coded from red indicating high signal intensity to green indicating low intensity and clustered using Pearson correlations. **c** Volcano plots showing the changes in PEs, TAGs, and PGs. These changes were grouped as PUFA (red fill) and SFA/MUFA (white fill). **d** Volcano plot showing changes in lipid profile in *SLC47A1*-knockdown cells versus control SW1990 cells. Red, upregulation; Green, downregulation; Black, no-significant. **e** When screened by the variable importance in the projection VIP ≥ 1, the volcano plot indicated the top 2 enriched lipid hits in *SLC47A1*-knockdown SW1990 cells. **f** Heatmap representing the relative abundances of free fatty acids in control and *SLC47A1*-knockdown SW1990 cells. Data are shown with three biologically independent samples. Elevated DHA and DPA were indicated by the red colour. **b**–**f** Differential-abundance analysis was performed between lipid species using two-tailed Student’s T-test (*P*-value <0.05).

SLC47A1 belongs to the multidrug and toxin extrusion protein (MATE) family and functions as an organic cation antiporter on the plasma membrane^31^. Since iron accumulation is the initial signal of ferroptosis resulting in the subsequent generation of reactive oxygen species (ROS)^32^, we wondered whether SLC47A1 affects intracellular iron accumulation. We found that the lack of *SLC47A1* had no significant effect on RSL3- or erastin-induced iron accumulation measured by means of the green-fluorescent heavy metal indicator Phen Green SK (Supplementary Fig. 3a). SLC47A1 is known to affect the intracellular delivery of drugs including metformin, cimetidine, and topotecan^31^. Therefore, we quantified the intracellular concentrations of RSL3 and erastin by liquid chromatography-mass spectrometry (LC-MS/MS). The silencing of *SLC47A1* failed to increase the intracellular concentrations of RSL3 or erastin (Supplementary Fig. 3b). Moreover, the knockdown of *SLC47A1* by shRNA did not affect the expression of known key ferroptosis regulators, such as GPX4 and ACSL4, with or without RSL3/erastin treatment (Fig. 3a). All these results indicate that SLC47A1 may regulate ferroptosis through a unique mechanism.

Since abnormal phospholipid transporters contribute to lipid remodeling under pathological conditions^33,34^, we next focused on the potential effects of SLC47A1 on intracellular lipid composition and metabolism through an untargeted lipidomic analysis. We used unsupervised principal component analysis (PCA score plot) to identify significant and reproducible effects of the SLC47A1 knockdown on the lipidomic profile (Supplementary Fig. 3c). The relative abundance of several lipid species was altered significantly in response to the knockdown of *SLC47A1* (Fig. 3b). Earlier studies reported that ferroptosis is executed by oxidized membrane phospholipids that contain PUFA chains^2,35^. Accordingly, the level of phosphatidyl-ethanolamines (PEs), triacylglycerols (TAGs), and phosphatidylcholines (PCs) that mainly contain PUFA chains were increased in *SLC47A1*-knockdown cells (Fig. 3c). Of note, PUFA-containing CEs were the most elevated class of lipids (Fig. 3d). Among CEs, when screened by variable importance in the projection (VIP) measure showing the importance of a variable in an orthogonal partial least-squares discriminant analysis, docosapolyenoic acid (DPA; C22:5) and docosahexaenoic acid (DHA; C22:6)-containing CEs were identified as the top 2 elevated lipids in *SLC47A1*-knockdown cells (Fig. 3e), highlighting a previously unrecognized metabolic role of SLC47A1 in regulating CE production.

**Fig. 4:**
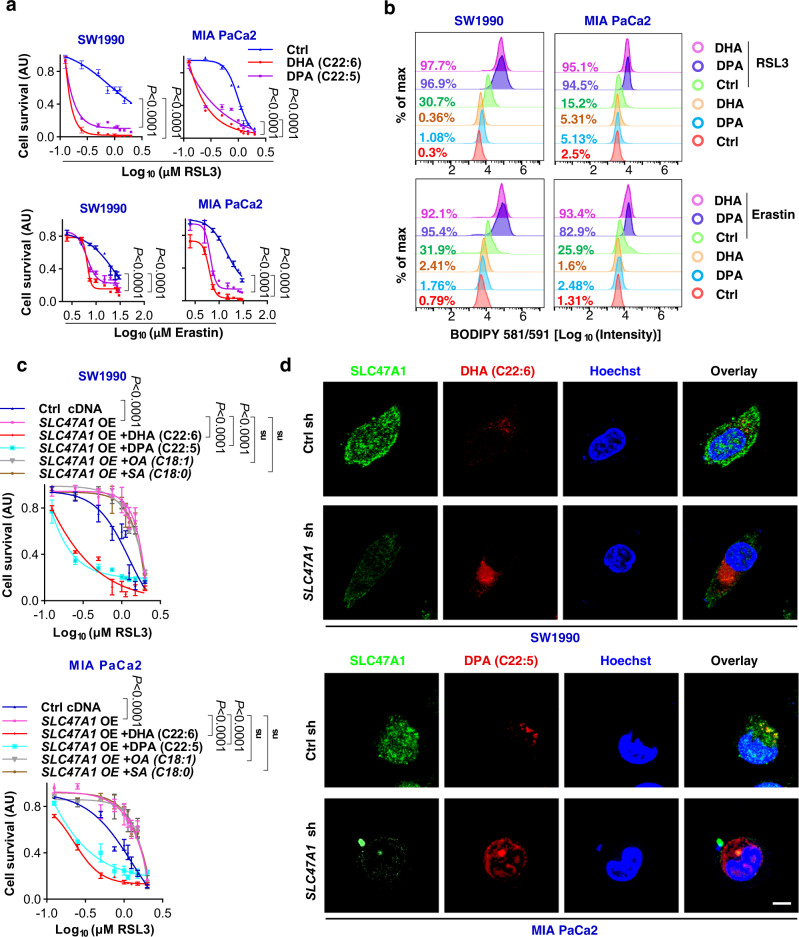
DHA/DPA contributes to the ferroptotic sensitivity in *SLC47A1*-depletion cells. **a** Indicated cells were pre-treated with DHA (20 μM) and DPA (20 μM) for 3 days, and treated with RSL3 or erastin for 24 h. Then cell viability were assayed. **b** Indicated cells were pre-treatment with DHA (20 μM) and DPA (20 μM) for 3 days, and treated with RSL3 or erastin for 6 h. Then lipid ROS were assayed. The data is representative of three independent experiments. Quantification results are provided in Supplementary Fig. 4. **c** Indicated cells were pre-treated with DHA (20 μM), DPA (20 μM), SA (20 μM) and OA (100 μM) for 3 days, and treated with RSL3 (1 μM) for 24 h. Then cell viability were assayed. **d** Representative images showing the co-localization of SLC47A1 (green) with DHA (red) or DPA (red) in SW1990 cells. The data is representative of three independent experiments. Scale bar, 10 µm. **a**, **c** Data are shown with 3 biologically independent samples, data were presented as mean ± SD, statistical significance was analyzed using two-way ANOVA with Dunnett’s multiple comparisons test. ns= not significant. Exact *p* values provided as source data. Source data are provided as a source data file.

In view of the major alteration of PUFA-esterified lipid metabolites in *SLC47A1*-knockdown cells, we hypothesized that SLC47A1 could be involved in the transport of fatty acids^36^. To test this hypothesis, we used gas chromatography-mass spectrometry (GC-MS) to quantify the abundance of medium- and long-chain fatty acids. In SW1990 cells, *SLC47A1* knockdown caused a massive increase in intracellular fatty acids, including DHA and DPA (Fig. 3f). Moreover, the administration of exogenous DHA and DPA sensitized PDAC cells to ferroptosis (Fig. 4a and Supplementary Fig. 4a) as well as lipid peroxidation (Fig. 4b and Supplementary Fig. 4b). In addition to DHA and DPA, monounsaturated fatty acid (MUFA) and saturated fatty acid (SFA) were also increased after *SLC47A1* knockdown. To determine whether MUFA or SFA are involved in the SLC47A1 pathway, we examined the effects of representative elevated MUFAs and SFAs, including exogenous oleic acid (OA, C18:1) and stearic acid (SA, C18:0), on ferroptosis in PDAC cells. OA increased resistance to ferroptosis, while SA sensitized cells to ferroptosis in WT cells (Supplementary Fig. 4c)^37,38^. Only exogenous DPA (C22:5) and DHA (C22:6), but not exogenous OA or SA, rescued ferroptosis sensitivity in SLC47A1-overexpressing cells (Fig. 4c).

As the lipid transport properties of SLC47A1, we explored the interaction between SLC47A1 and DHA/DPA. Molecular docking study showed that both DHA and DPA had binding affinity to SLC47A1 and could enter the pocket-like structure of SLC47A1 (Supplementary Fig. 4d). Further immunofluorescence analysis confirmed the co-localization of SLC47A1 and DHA/DPA in SW1990 cells (Fig. 4d). Functionally, substantial enrichment of intracellular DHA/DPA was observed in *SLC47A1*-knockdown cells (Fig. 4d). These findings support a direct role for SLC47A1 in regulating DHA/DPA accumulation during ferroptosis.

### The ACSL4-SOAT1 axis mediates enhanced ferroptosis sensitivity caused by SLC47A1 deficiency

**Fig. 5:**
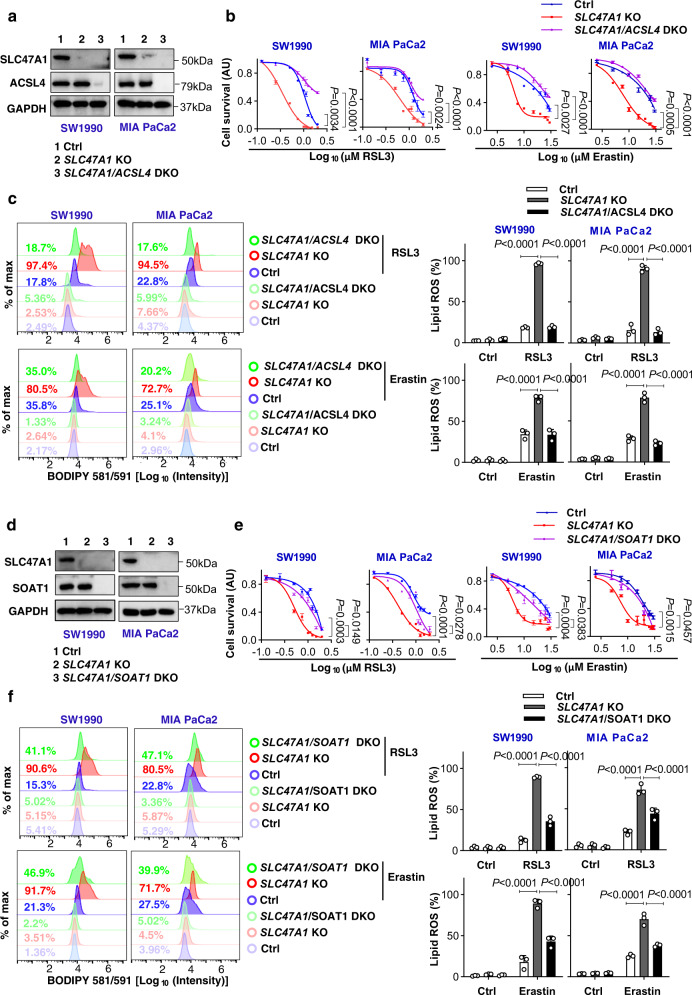
The ACSL4-SOAT1 axis mediates enhanced ferroptosis sensitivity caused by SLC47A1 deficiency. **a** Western blot analysis of protein expression in indicated SW1990 and MIA PaCa2 cells. The data is representative of three independent experiments. **b** Cell viability of indicated cells following treatment with RSL3 or erastin for 24 h. **c** Indicated cells were treated with RSL3 (1 μM) or erastin (10 μM) for 6 h, and then lipid ROS was measured. The data is representative of three independent experiments. **d** Western blot analysis of protein expression in indicated SW1990 and MIA PaCa2 cells. The data is representative of three independent experiments. **e** Cell viability of indicated cells following treatment with RSL3 or erastin for 24 h). **f** Indicated cells were treated with RSL3 (1 μM) or erastin (10 μM) for 6 h, and then lipid ROS was measured. The data is representative of three independent experiments. **b**, **e** Data are shown with 3 biologically independent samples, data were presented as mean ± SD, and statistical significance was analyzed using two-way ANOVA with Dunnett’s multiple comparisons test. **c**, **f** The data is representative of three independent experiments, data were presented as mean ± SD, statistical significance was analyzed using one-way ANOVA with Dunnett’s multiple comparisons test. Exact *p* values are provided as source data. Source data are provided as a source data file. KO, knockout; DKO: double knockout.

ACSL4 is responsible for the esterification of coenzyme A (CoA) to free fatty acids and acts as a biomarker as well as a driver of ferroptosis^8–10^. The knockdown of *SLC47A1* by shRNA had no effects on erastin- or RSL3-induced ACSL4 upregulation (Fig. 3a). To determine whether basic ACSL4 expression is required for the loss of *SLC47A1*-induced sensitization to ferroptosis, we compared the phenotype of wild type, *SLC47A1*-knockout, and *SLC47A1/ACSL4* double-knockout cells generated by CRISPR-Cas9 technology (Fig. 5a). Like the knockdown of *SLC47A1* by shRNA (Fig. 3a), the knockout of *SLC47A1* by CRISPR-Cas9 did not affect the basic expression of ACSL4 (Fig. 5a). However, compared with *SLC47A1*-knockout cells that were sensitive to RSL3- and erastin-induced growth inhibition, cell death, and lipid peroxidation, *SLC47A1/ACSL4* double-knockout cells were relatively resistant to ferroptosis induction (Fig. 5b, c and Supplementary Fig. S5a). These data indicate that baseline expression of ACSL4 is required for the sensitization to ferroptosis caused by SLC47A1 deficiency.

PUFAs that are esterified to complex lipids are especially susceptible to hydrogen abstraction by reactive radicals, initiating the lipid peroxidation chain reaction^39^. Lysophosphatidylcholine acyltransferases (LPCATs) are a group of enzymes responsible for phospholipid remodeling that modulate the fatty acyl composition of phospholipids^40^. After ligating CoA through ACSL4, long-chain PUFAs are used to esterify lysophospholipids through LPCAT3, which promotes ferroptosis^8,41^. We confirmed that LPCAT3-deficiency alone conferred ferroptosis resistance to PDAC cells (Supplementary Fig. S5b, c). However, the silencing of *LPCAT3* failed to reverse ferroptotic cell death in SLC47A1-knockdout cells (Supplementary Fig. S5d, e), indicating that when SLC47A1 is inhibited, ferroptosis does not require LPCAT3.

Under oxidative stress, both PUFA-containing phospholipids and CEs can be oxidized through a free radical-induced lipid peroxidation process to form a complex mixture of oxidation products^42,43^. Since PUFA-CE was enriched in *SLC47A1*-knockdown cells (Fig. 3e), we investigated the effect of downstream mediators of ACSL4 activation on PUFA-CE production in *SLC47A1*-inhibited cells. We focused on sterol O-acyltransferase 1 (SOAT1, also known as ACAT1), which is a rate-limiting enzyme that catalyzes cholesterol and fatty acids into CEs^44,45^. We suppressed *SOAT1* expression in SW1990 and MIA PaCa2 cells by two different siRNAs (Supplementary Fig. S5f). The silencing of *SOAT1* alone had no effects on ferroptosis sensitivity in SLC47A1-sufficient cells (Supplementary Fig. S5g), but the knockout of SOAT1 inhibited erastin- or RSL3-induced growth inhibition and lipid peroxidation in *SLC47A1*-knockout cells (Fig. 5d–f). However, the inhibition of *ACSL4* suppressed the anticancer activity of erastin or RSL3 in both wild-type and *SLC47A1*-knockout cells (Fig. 5a, b and Supplementary Fig. 5h, i). These findings support the hypothesis that ACSL4 contributes to lipid peroxidation in both wild-type and *SLC47A1*-knockout cells, while SOAT1 is selectively used for ferroptosis in *SLC47A1*-knockout cells, commensurate with the fact that SLC47A1 depletion leads to PUFA-CE accumulation.

### PPARA is required for SLC47A1 upregulation during ferroptosis

**Fig. 6:**
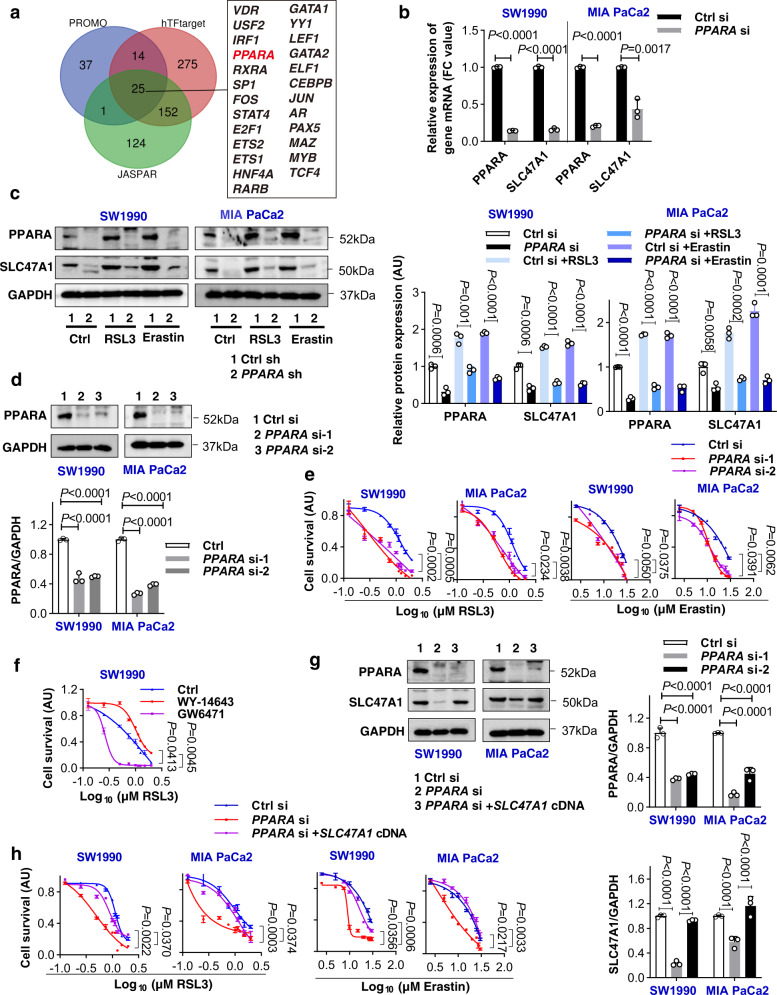
PPARA is required for SLC47A1 upregulation during ferroptosis. **a** Venn diagram showing the putative upstream TFs of SLC47A1 predicted by JASPAR, PROMO, and hTFtarget databases. **b** qPCR analysis of *PPARA* and *SLC47A1* mRNA in indicated wild type and *PPARA*-knockdown cells. **c** Western blot analysis of protein expression in indicated cells following treatment with RSL3 (1 μM) and erastin (10 μM) for 24 h. **d** Western blot analysis of protein expression in indicated wild type and *PPARA*-knockdown cells. **e** Cell viability of indicated cells following treatment with RSL3 or erastin for 24 h. **f** Cell viability of SW1990 following treatment with RSL3 (1 μM, 24 h) when PPARα activity was modulated using an agonist (WY-14643, 10 μM) or an antagonist (GW6471, 10 μM). **g** Western blot analysis of protein expression in indicated wide type and *PPARA*-knockdown cells with or without the transfection of *SLC47A1* cDNA. **h** Cell viability of indicated cells following treatment with RSL3 or erastin for 24 h. **b**–**h** Data are shown with 3 biologically independent samples, data were presented as mean ± SD, statistical significance was analyzed using unpaired two-tailed T-test (**b**, **c**), one-way ANOVA (**d**, **g**) or two-way ANOVA (**e**, **f**, **h**) with Dunnett’s multiple comparisons test. Exact *p* values are provided as source data. Source data are provided as a source data file.

To identify the upstream transcription factor (TF) that controls the expression of SLC47A1, we took advantage of the publicly accessible databases JASPAR (http://jaspardev.genereg.net/), PROMO (http://alggen.lsi.upc.es), and hTFtarget (http://bioinfo.life.hust.edu.cn/hTFtarget). Using these sites for a bioinformatics analysis, we identified 25 candidate TFs that may bind to the *SLC47A1* promoter (Fig. 6a). Among them, PPARA is a key TF in the large scale rewiring of lipid homeostasis^46,47^, which has already been reported to transactivate *SLC47A1* in mouse kidneys^48^. To determine whether PPARA plays a similar role in regulating the expression of SLC47A1 in ferroptosis, we suppressed PPARA expression by siRNAs in SW1990 and MIA PaCa2 cells. Western blots and qPCR analysis indicated that, the knockdown of *PPARA* inhibited the protein and mRNA expression of SLC47A1 both in the absence and presence of RSL3 or erastin (Fig. 6b and c). Thus, PPARA is involved in the basic expression of SLC47A1, as well as in its ferroptosis-associated upregulation. Accordingly, we observed an increase in PPARA protein levels in response to RSL3 and erastin (Fig. 6c), supporting that PPARA is a TF that can be induced by stress^46,47^.

Next, we examined whether the knockdown of *PPARA* induces a similar functional phenotype as *SLC47A1*-knockdown cells with respect to ferroptosis susceptibility. Like the *SLC47A1*-knockdown cells, *PPARA*-knockdown SW1990 and MIA PaCa2 cells were sensitive to RSL3- or erastin-induced growth inhibition and cell death (Fig. 6d, e and Supplementary Fig. S6a). Ferrostatin-1 fully blocked RSL3- or erastin-induced cell death in *PPARA*-knockdown cells (Supplementary Fig. S6a). Lipid peroxidation analysis further confirmed the hypothesis that PPARA acts as a negative regulator of ferroptosis (Supplementary Fig. S6b). Consistent with experimental conclusions using PPARA siRNA, the PPARA agonist (WY-14643) inhibited ferroptosis, while the PPARA antagonist (GW6471) increased ferroptosis sensitivity (Fig. 6f).

To determine whether PPARA-mediated SLC47A1 expression is required for PPARA-mediated ferroptosis resistance, *SLC47A1* was overexpressed in *PPARA*-knockdown cells (Fig. 6g). This rescue experiment demonstrated that transfection-enforced re-expression of *SLC47A1* restores resistance to ferroptosis in *PPARA*-knockdown SW1990 and MIA PaCa2 cells (Fig. 6h). Altogether, these findings establish that PPARA-dependent SLC47A1 expression attenuates the susceptibility of cells to ferroptosis induction.

### The PPARA–SL47A1 pathway limits ferroptosis in vivo

**Fig. 7:**
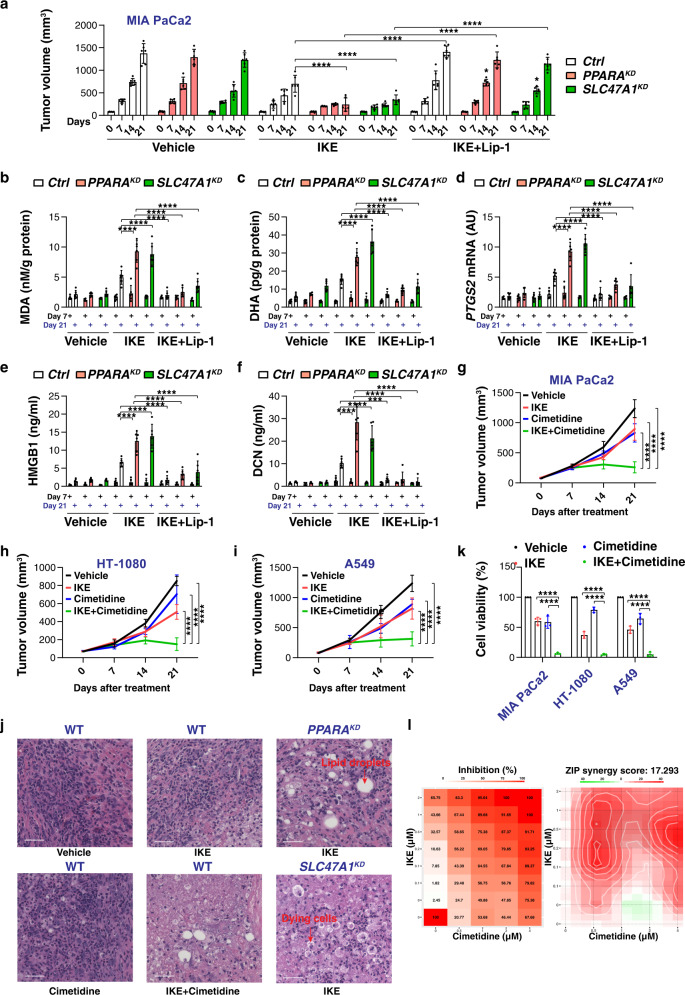
The PPARA-SLC47A1 pathway limits ferroptotic cancer cell death in vivo. **a** Athymic nude mice were injected subcutaneously with indicated *PPARA*-knockdown (*PPARA*^*KD*^) or *SLC47A1*-knockdown (*SLC47A1*^*KD*^) MIA PaCa2 cells for 7 days and then treated with IKE (20 mg/kg, i.p., once every other day) in the absence or presence of liproxstatin-1 (Lip-1; 10 mg/kg, i.p., once every other day) at day 7 for 2 weeks. Tumor volumes were calculated weekly (*n* = 6 mice/group; ****p < 0.0001, two-way ANOVA with Tukey’s multiple comparisons test; data are presented as mean ± SD). The abscissa shows the time after drug treatment. **b**–**f** In parallel, the levels of MDA, DHA, or *PTGS2* mRNA in isolated tumors and serum HMGB1 and DCN at day 7 and day 21 after treatment were assayed (*n* = 6 mice/group; two-way ANOVA with Tukey’s multiple comparisons test; data are presented as mean ± SD). **g**–**i** Athymic nude mice were injected subcutaneously with MIA PaCa2, HT-1080, or A549 cells for 7 days and then treated with IKE (20 mg/kg, i.p., once every other day) in the absence or presence of cimetidine (20 mg/kg, i.p., once every other day) at day 7 for 2 weeks. Tumor volumes were calculated weekly (*n* = 6 mice/group; two-way ANOVA with Tukey’s multiple comparisons test; data are presented as mean ± SD. **j** Representative H&E stained sections in the MIA PaCa2 xenograft model at day 21 (bar = 100 µm). **k** Viability of indicated cells following treatment with IKE (2 μM) and/or cimetidine (1 μM) for 24 h (*n* = 3 biologically independent samples, two-way ANOVA with Tukey’s multiple comparisons test; data are presented as mean ± SD). **l** MIA PaCa2 cells were treated with the indicated doses of IKE and cimetidine for 24 h, and relative cell viability was assessed. Shown is the calculation and visualization of ZIP synergy scores for the drug combination of IKE and cimetidine performed by SynergyFinder. The growth inhibition (%) in the dose-response matrix of the indicated cell lines are shown. A score ≥10 indicates a significant synergistic effect. Exact *p* values are provided as source data. Source data are provided as a source data file.

We next used xenograft mouse models to investigate whether inhibiting the PPARA-SL47A1 pathway enhances the anticancer activity of imidazole ketone erastin (IKE), a metabolically stable analog of erastin used in vivo^49^. Consistent with previous studies^50^, a xenograft model derived from human MIA PaCa2 cells was an aggressive animal model with accelerated tumor growth (Fig. 7a**)**. In line with our in vitro studies, *PPARA*-knockdown (*PPARA*^*KD*^) or *SLC47A1*-knockdown (*SLC47A1*^*KD*^) MIA PaCa2 cells were more sensitive to IKE-induced tumor suppression compared to control groups in vivo (Fig. 7a). This increased antitumor activity of IKE in *PPARA*^*KD*^ and *SLC47A1*^*KD*^ cells was reversed by the ferroptosis inhibitor liproxstatin-1 (Fig. 7a). Subsequent quantitation of the intratumoral levels of MDA, DHA, mRNA of prostaglandin-endoperoxide synthase 2 (*PTGS2*, a biomarker of ferroptosis in vivo^5^), and circulating levels of high mobility group box 1 (HMGB1, an indicator of cell death^51^) and proteoglycan decorin (DCN, a specific marker for ferroptosis, but not for other cell deaths^52^) at endpoint day 21 confirmed that PPARA and SLC47A1 depletion sensitizes cells to ferroptosis (Fig. 7b–f). However, we did not observe significant differences in these markers between the control, *PPARA*^*KD*^ and *SLC47A1*^*KD*^ groups with or without IKE treatment on day 7 (Fig. 7b–f). This was consistent with previous findings that the tumor suppressor activity of IKE and induction of ferroptosis in xenograft models require repeated administration to obtain^49^.

Cimetidine, a potential SLC47A1 inhibitor^53^, also increased IKE-induced tumor suppression in xenograft mouse models involving the implantation of MIA PaCa2, HT-1080 (a human fibrosarcoma cell line), or A549 (human lung carcinoma cell line) cells (Fig. 7g–i). Like the knockdown of *PPARA* or *SLC47A1*, hematoxylin and eosin (H&E) staining observed that the combination of IKE and cimetidine resulted in increased necrotic-like changes in tumor tissue compared to the treatment group alone (Fig. 7j). In vitro, cimetidine also increased IKE-induced growth inhibition in MIA PaCa2, HT-1080, or A549 cells (Fig. 7k). SynergyFinder, a standalone web-application for analyzing and visualizing multidrug combination response data^54^, confirmed the synergistic anticancer effect of IKE and cimetidine in MIA PaCa2 cells (Fig. 7l).

## Discussion

**Fig. 8:**
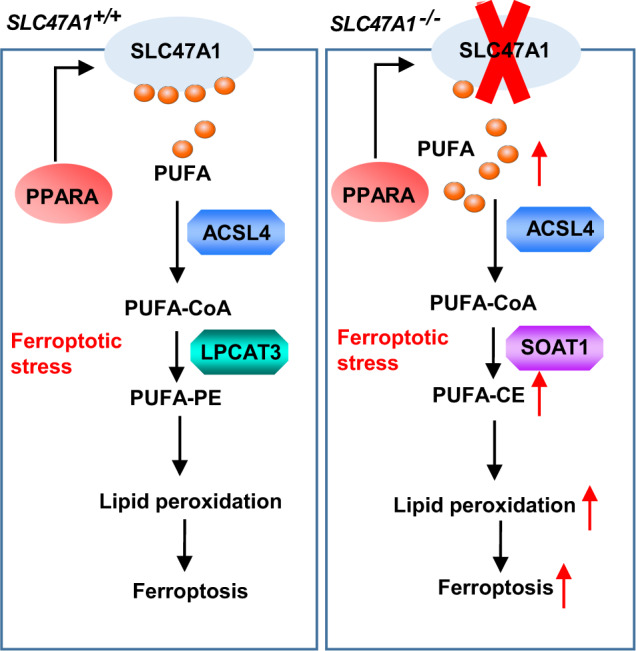
A model illustrating the metabolic role of SLC47A1 in inhibiting ferroptosis. PPARA-mediated expression of lipid flipase SLC47A1 on cell membranes is a regulator of lipid remodeling and survival during ferroptosis. In wild type cells, the ACSL4-SOAT1 pathway-mediated PUFA-CE production favors ferroptosis. In SLC47A1 deficient cells, the ACSL4-SOAT1 pathway-mediated PUFA-CE production promotes ferroptosis.

The physicochemical properties of cell membranes have a major impact on the balance between cell survival and death^55,56^. Sensitivity to ferroptosis is determined by lipid remodeling, which provides substrates for lethal lipid peroxidation^7^. Thus, the phospholipids PE and PC play a major role in mediating ferroptosis^9,57^. Here, we report a previously unrecognized mechanism through which the lipid flippase SLC47A1 acts as an endogenous repressor of ferroptosis, presumably by affecting the concentrations of PUFAs as well as their metabolism (Fig. 8). Moreover, our observations suggest that the direct inhibition of SLC47A1 (by its knockdown, knockout, or by means of cimetidine) or its indirect inhibition (by the knockdown of PPARA) can sensitize cancer cells to pharmacological ferroptosis induction.

In 2005, two human orthologs of the bacterial MATE family transporters were cloned and are now officially named SLC47A1 (hMATE1) and SLC47A2 (hMATE2), respectively^31^. SLC47A1 is expressed throughout the body (including the pancreas), while SLC47A2 is specific for the kidney^31^. MATE-type transporters are known to be involved in the excretion of various cationic drugs^58^, but their role in cell death pathways remains poorly understood. Here, we observed that SLC47A1 is selectively upregulated by inducers of ferroptosis (RSL3- or erastin) but not apoptosis (staurosporin). However, SLC47A1 does not modulate the intracellular concentration of the ferroptosis inducers. As a transmembrane transporter that flips lipids, SLC47A1 might transport lipids from the outer to the inner leaflet of the bilayer. Indeed, untargeted lipidomic analysis revealed that SLC47A1 depletion increased the intracellular abundance in PUFA-esterified CE, thereby promoting ferroptosis. These findings not only highlight alternative pathways involving CE-mediated ferroptosis, but also establish an anti-ferroptotic defense mechanism that relies on SLC47A1-mediated lipid transport. The relationship between the SLC47A1 pathway and other membrane defense mechanisms (such as ESCRT-III-mediated membrane repair^28,29^ and the activation of the enzymes that reduce lipid peroxides^59^) requires further investigation.

Our study strengthens the evidence for the pro-ferroptotic role of ACSL4. In 2016, three independent groups, including ours, used an array of different methods to identify ACSL4 as a biomarker and mediator of ferroptosis^8–10^. ACSL4 links free PUFAs to CoA, generating fatty acyl-CoA esters, which are eventually incorporated into PC or PE by LPCAT3. Pro-ferroptotic PUFA-PC/PE production is usually mediated by the ACSL4-LPCAT3 pathway^8,9^. However, in the context of SLC47A1 deficiency, ferroptosis instead PUFA-CE production is mediated by the alternative ACSL4-SOAT1 pathway. In this sense, SLC47A1 may be regarded as a metabolic checkpoint that shifts PUFA metabolism into distinct directions^60^.

Our findings also suggest a mechanism that links the well-established toxicity of excessive cholesterol^61,62^ to ferroptosis. In addition to phospholipids, the plasma membrane of mammalian cells contains cholesterol (about 30% of total lipids), which affects membrane fluidity^63^. Membranes containing a large amount of DHA have high permeability, a high lipid flip-flop rate, and free radical propagation ability^64,65^. The inert characteristics of CEs is dramatically altered if cholesterol is esterified to PUFAs and subjected to oxidative modification^66^. Recent studies have shown that n-3 and n-6 long-chain PUFAs that exceed the storage capacity of lipid droplets can be peroxidized and will then induce ferroptotic cancer cell death^62^. Our study indicates that SLC47A1 inhibits the generation of PUFA-CE and subsequent lipid peroxidation during ferroptosis.

Our results indicate that PPARA is responsible for SLC47A1 expression. PPARA is a selective intracellular fatty acid sensor that regulates lipid remodeling by transactivating multiple genes involved in lipid metabolism^46,47^. PPARA activity is regulated by MDM2 and its homolog MDMX, which limits ferroptosis^67^. It will be interesting to determine the extent that genetic or pharmacological inhibition of PPARA-dependent SLC47A1 expression enhances ferroptosis-mediated tumor suppression in vivo. Metabolic reprogramming is crucial for cancer cells to adapt to a hostile tumor microenvironment and ultimately become metastatic^68^ and it will be important to investigate the extent to which SLC47A1 and other lipid transporters may be involved in tumor progression^22^.

A key limitation of our study is that the subcutaneous xenograft tumor models used are often not representative of the human tumor microenvironment, and so far these models have not predicted treatment success as hoped. Therefore, patient-driven tumor models combined with transgenic animal models are needed to further confirm whether inhibition of the PPARA-SL47A1 pathway is a potential strategy to enhance ferroptosis-mediated tumor suppression. Since ferroptosis also occurs in normal tissues^69^, the use of IKE may cause potential side effects, which may require the development of tissue- or cell-dependent ferroptosis activators for clinical trials^4^. Although inhibition of SLC47A1 activity by cimetidine was well documented in vitro^53^, there is currently no published method to assess the activity of SLC47A1 in tissues. There is also an urgent need to develop potent and low-toxic SLC47A1 inhibitors and simple assays for assessing SLC47A1 activity for translational research.

In summary, our study suggests that SLC47A1 plays an important role in determining ferroptosis resistance through a unique mechanism that relies on the rewiring of PUFA metabolism. Importantly, SLC47A1 constitutes an actionable target for sensitizing pancreatic cancer cells to the pharmacological induction of ferroptosis^70,71^.

## Methods

The reagents are described in Supplementary Data 1.

### Cell culture and treatment

SW1990 (CRL-2172), MIA PaCa2 (CRL-1420), HT-1080 (CCL-121), MCF-7(HTB-22), SKOV3 (HTB-77), and A549 (CCL-185) cell lines were obtained from the American Type Culture Collection. An NALM-6 (FH1161) cell line was obtained from Fu Heng Biology. These cell lines were grown in Dulbecco’s modified Eagle’s medium (Thermo Fisher Scientific, 11995073) or RPMI-1640 medium (Thermo Fisher Scientific, 22400097) with 10% fetal bovine serum (Thermo Fisher Scientific, A3840001) and 1% penicillin and streptomycin (Thermo Fisher Scientific, 15070-063) at 37 °C, 95% humidity, and 5% CO_2_. All cell lines were mycoplasma-negative and characterized by short tandem repeat profiling. Dimethyl sulfoxide (DMSO) was used to prepare the stock solution of drugs. The final concentration of DMSO in the drug-working solution in the cells was <0.01%. DMSO of 0.01% was used as vehicle control in all cell culture assays.

### Cell viability assay

The level of cell viability was assayed using a Cell Counting Kit-8 (CCK-8) kit (YEASEN, 40203ES80) according to the manufacturer’s protocol. Cells were seeded in 96-well plates (1 × 10^4^ cells/well) and treated with erastin or RSL3 for 24 h. For each well of the plate, the medium was replaced with 100 µl of fresh medium containing 10 µl (or directly added in the NALM-6 cell line) of CCK8 solutions. The culture was then returned to the cell incubator for 60–90 min. Absorbance at 450 nm was proportional to the number of living cells in the culture. Cell viability was expressed as a relative level, and 100% cell viability was set as 1.

### Cell death assay

Cells were seeded at a density of 2 × 10^5^ cells/well in medium in 6-well plates. The next day, cells were incubated with the indicated treatments. After that, the cells were stained with propidium iodide (BestBio, BB-4131-1) for 20–30 min in an incubator of 5% CO_2_ at 37 °C. Morphological changes were examined using fluorescence microscope at ×100 magnification. A Countess II FL Automated Cell Counter (Thermo Fisher Scientific) was used to assay the percentages of dead cells after a propidium iodide staining. The cell death is expressed as a relative level, and 100% cell death is set to 1.

### Iron staining

The metal sensor Phen Green SK diacetate (Thermo Fisher Scientific, P14313) was used to detect intracellular labile iron^72^. Phen Green SK is a probe containing a fluorescein (dichlorofluoresceinamine) and a metal binding moiety (phenanthroline). The degree to which Phen Green SK fluorescence was quenched gave an estimate of the amounts of cellular cheatable iron. Cells were seeded at a density of 2 × 10^5^ cells/well in medium in 6-well plates. The next day, cells were incubated with the indicated treatments. After that, the cells were then stained with 2.5 µM Phen Green SK for 20–30 min in an incubator of 5% CO_2_ at 37 °C. Then fluorescence changes were examined by fluorescence microscope at ×200 magnification.

### Lipid ROS assay

BODIPY 581/591 C11 probe (Thermo Fisher Scientific, D3861) was used to detect lipid ROS according to the manufacturer instructions^73^. In brief, cells were incubated with BODIPY 581/591 C11 at a final concentration of 5 µM for 30 min at 37 °C and washed three times with PBS. Oxidation of the polyunsaturated butadienyl portion of the dye resulted in a shift of the fluorescence emission peak from 590 nm to 510 nm, which was measured using a BD Accuri C6 Plus flow cytometer (BD Biosciences). A minimum of 10,000 cells were analyzed for each condition.

### RNAi and plasmid transfection

Transfection with siRNA, shRNA, and cDNA was performed using Lipofectamine RNAiMAX (Thermo Fisher Scientific, 13778-030) or lipofectamine 3000 (Thermo Fisher Scientific, L3000-015) according to the manufacturer’s instructions. We used 293FT cells (Thermo Fisher Scientific, R70007) to produce high-titer lentiviral particles, and the virus-containing medium was harvested 48 h after transfection. In addition, 2 μg/ml puromycin (YEASEN, 60210ES72) was used for the selection of transduced cells. The expression efficiency was evaluated by qPCR or western blot analysis. The sequence or order information of shRNA, and cDNA are shown in Supplementary Data 1. The sequence of a pooled siRNA library targeting phospholipid transporter genes is shown in Supplementary Data 2.

### CRISPR-Cas9 gene editing

Specific sgRNAs targeting the indicated genes were designed using online software from Feng Zhang lab (https://zlab.bio/guide-design-resources). sgRNAs were cloned into the LentiCRISPR V2 vector. Lentiviruses were generated from sgRNA constructs in 293FT packaging cells in a 6-well plate format using lipofectamine 3000 (Thermo Fisher Scientific, L3000-015) as transfection reagent and infected cells for sgRNA expression^74^. Infected cells were selected with 2 μg/mL of puromycin starting 48 h post-infection and propagated for further single-cell cloning. Transfected cells were sorted into 96-well plates at 1 cell/well. Cells were grown for 7 days to become single-cell clones. The sequences of sgRNA oligonucleotides and primers are described in Supplementary Data 3.

### qPCR assay

Total RNA was extracted using a QIAGENan RNeasy Plus Micro Kit (QIAGEN, 74034) according to the manufacturer’s instructions. Cell lysates were spun through using the kit’s gDNA Eliminator spin columns to remove genomic DNA. Total RNA was purified using RNeasy MinElute spin columns. First-strand cDNA was then synthesized from 1 µg of RNA using the PrimeScript™ RT Master Mix (Takara, RR036A). Then 20 µl reactions were prepared by combining 4 µl of PrimeScript™ RT Master reaction mix, 2 µl of gene-specific enhancer solution, 1 µl of reverse transcriptase, 1 µl of gene-specific assay pool (20×, 2 µM), and 12 µl of RNA diluted in RNase-, DNase-, and genomic DNA-free water. Quantitative real-time PCR was performed qPCR using TB Green Premix Ex Taq II (Takara, RR820Q) on the C1000 Touch Thermocycler CFX96 Real-Time System (Bio-Rad). Analysis was performed using Bio-Rad CFX Manager software 2.0 (Bio-Rad). The data were normalized to RNA GAPDH and the fold change was calculated via the 2-DDCt method. The relative concentrations of mRNA were expressed in arbitrary units based on the untreated group, which was assigned a value of 1. The primers, which were synthesized and desalted, were from Beijing Genomics Institution, and are shown in Supplementary Data 4.

### Western blot

Cells were lysed 1× in cell lysis buffer (Cell Signaling Technology, 9803) with protease and phosphatase inhibitor cocktail (Cell Signaling Technology, 5872) on ice for 30 min^75^. After centrifugation at 14,000 × *g* for 15 min at 4 °C, the supernatants were collected and quantified using a bicinchoninic acid (BCA) assay (Thermo Fisher Scientific, 23225). The 30 µg proteins were separated on 10% or 12.5% polyacrylamide gel electrophoresis (PAGE) gels (Epizyme, PG112, PG113) and transferred to polyvinylidene difluoride (PVDF) membranes (Millipore, IPVH00010). Following blocking by TBST containing 5% skim milk for 1 h, the membrane was incubated overnight at 4 °C with various primary antibodies (1:200-1:1000). After incubation with peroxidase-conjugated secondary antibodies (rabbit anti-goat IgG secondary antibody [Abcam, ab6741; 1:1000]) for 1 h at room temperature, the signals were visualized using enhanced chemiluminescence (Thermo Fisher Scientific, 34095). Blots were analyzed using the ChemiDoc Touch Imaging System (Bio-Rad) and Image Lab Software (Bio-Rad). The information on antibodies is shown in Supplementary Data 1.

### LC-MS/MS for lipid analysis

Lipids were extracted according to MTBE method. Briefly, samples were homogenized with 200 µl water and 240 µl methanol. Then 800 µl of MTBE was added and the mixture was exposed to ultrasound for 20 min at 4 °C followed by sitting still for 30 min at room temperature. The solution was centrifuged at 14,000 × *g* for 15 min at 10 °C and the upper organic solvent layer was obtained and dried under nitrogen. Reverse phase chromatography was selected for LC separation using CSH C18 column (1.7 µm, 2.1 mm × 100 mm, Waters). The lipid extracts were re-dissolved in 200 µl of 90% isopropanol/ acetonitrile, centrifuged at 14,000 × *g* for 15 min, and finally 3 µl of sample was injected. Solvent A was acetonitrile–water (6:4, v/v) with 0.1% formic acid and 0.1 mM ammonium formate, and solvent B was acetonitrile–isopropanol (1:9, v/v) with 0.1% formic acid and 0.1 mM ammonium formate. The initial mobile phase was consisted of 30% solvent B at a flow rate of 300 µl/min. It was held for 2 min, and then linearly increased to 100% solvent B in 23 min, followed by equilibrating at 5% solvent B for 10 min. Mass spectraspectrometry was acquired by performed using a Q-Exactive Plus spectrometer (Thermo Fisher Scientific) in positive and negative mode, respectively^76^.

### Lipid data processing analysis

Lipid species were identified using the LipidSearch software version 4.2 (Thermo Scientific) to process the raw data and for peak alignment, retention time correction, and extraction peak area. Adducts of +H, + NH4 were selected for positive mode searches, whereas -H, + CH3COO were selected for negative mode searches because ammonium acetate was used in the mobile phases. For data extracted from LipidSearch, ion peaks with >50% missing values in the group were removed. The internal standards for different lipid species identification are provided in Supplementary Data 5. After normalization and integration using the Perato scaling method, the processed data were imported into SIMPCA-P 16.1 (Umetrics, Umea, Sweden) for multivariate statistical analysis, including principal component analysis (PCA), partial least squares discriminant analysis (PLS-DA), and orthogonal partial least squares discriminant analysis (OPLS-DA). Lipids with significant differences were identified based on a combination of statistically significant thresholds of variable influence on projection (VIP > 1) values obtained from the OPLS-DA model (mutidi-mensional statistical analysis) and two-tailed student’s t-test (P < 0.05) on the raw data (unidimensional statistical analysis) by PASS 16 (https://www.ncss.com/software/pass/) before experiments. Median-normalized lipidomic datasets are presented in Supplementary Data 6.

### GC-MS for fatty acid analysis

The cell samples were thawed on ice, and 50 µl of each sample was added into a 2 ml glass centrifuge tube. Then, 1 ml of chloroform-methanol (2:1 v/v) was added. After ultrasonication for 30 min, 2 ml of 1% sulfuric acid in methanol was added to the supernatant. The mixture was incubated in an 80 °C water bath for 30 min to achieve fatty-acid esterification for methyl esterification. Then, 1 ml n-hexane and 5 ml water were added, and vortex mixed. The supernatant (500 µl) was spiked with an internal standard (25 µl of 500 ppm methyl salicylate), mixed, and subjected to GS-MS using an Agilent Model 7890 A/5975 C GC-MS system. To quantify medium- and long-chain fatty acids, supelco 37-component FAME (fatty-acid methyl ester) mix (Sigma-Aldrich) was used to construct a calibration curve for the concentration range of 0.5–1000 mg/l. The IS was used to correct for injection variability between samples and minor changes in the instrument response. The samples were separated with an Agilent DB-WAX capillary GC column (30 m × 0.25 mm ID × 0.25 µm). The initial temperature was 50 °C and remained as such for 3 min. The temperature was then increased to 220 °C at 10 °C/min, and remained at 220 °C for 20 min. The carrier gas was helium (1.0 mL/min). A quality-control sample was used for testing and evaluating the stability and repeatability of the system.

### Fatty acid data processing analysis

The MSD Chem Station software was used to extract chromatographic peak area and retention time. The content of medium and long chain fatty acids in the sample was calculated by plotting the curve. The quality control samples were processed together with the biological samples. Detected metabolites in pooled samples with coefficient of variation (CV) less than 30 % were denoted as reproducible measurements. To find different expressed metabolites, statistical analyses between two sample groups were performed by calculating the Student’t test *p* values of metabolites. Metabolites with *P* values <0.05 were marked as the significantly changed metabolites between sample groups. Median-normalized fatty acid datasets are presented in Supplementary Data 7.

### LC-MS/MS for intracellular drug concentration detection

The cell samples were thawed on ice, vortexed, and freeze-thawed three times and vortexed to completely break the cells. Then we took a 100 µl sample and added 10 L internal standard, vortexed for 10 s, added 500 L ethyl acetate, and vortexed for 30 s. After concentration centrifugation at 14,000 × *g* for 5 min, 400 µl of supernatant was collected for LC-MS/MS analysis. A NexeraX2 LC-30AD (Shimadzu) chromatograph system coupled with a 3200 QTRAP mass spectrometer (Sciex) was used for quantitative analysis. An Atlantis T3 C18 column (50 mm × 2.1 mm I.D.3.5 µm) was used and the column oven temperature was 40 °C. The mobile phase was composed of solvent A (H_2_O) and B (acetonitrile), both containing 0.1 % formic acid. The gradient was as follows: 0–1 min, 5% B; 1–1.2 min, 5% B; 1.2–3.0 min, 95% B; 3.0–3.2 min, 95% B; and 3.2–4.0 min, 5% B for column re-equilibration.

### Animal model

We conducted all animal care and experiments in accordance with the Association for Assessment and Accreditation of Laboratory Animal Care guidelines and with approval from our institutional animal care and use committees (Central South University). The maximal tumor size/burden permitted in the protocol of the institutional animal care and use committees did not exceed 2000 mm^3^. All mice were housed under a 12-h light-dark diurnal cycle with controlled temperature (20 °C–25 °C) and relative humidity (40–60%). Food (Laboratory Rodent Diet, LabDiet, 5001) and water were available *ad libitum*. Experiments were carried out under pathogen-free conditions and the health status of mouse lines was routinely checked by veterinary staff. Experiments were carried out with randomly chosen littermates of the same sex and matched by age and body weight. Animals were sacrificed at the indicated time by CO_2_ asphyxia, and blood samples and tissue were collected.

To generate murine subcutaneous tumors, 5 × 10^6^ indicated human tumor cells in 50 μl PBS were injected subcutaneously into the right of the dorsal midline in 6- to 8-week-old athymic nude female mice (Charles River). Once the tumors reached 60–90 mm^3^ at day 7, mice were randomly allocated into groups and then treated with IKE (40 mg/kg, i.p., once every other day) in the absence or presence of liproxstatin-1 (10 mg/kg, i.p., once every other day) or cimetidine (20 mg/kg, i.p., once every other day) at day 7 for 2 weeks. Tumors were measured twice weekly, and volumes were calculated using the formula length × width^2^ × π/6.

### Enzyme-linked immunosorbent assay

Commercially available enzyme-linked immunosorbent assay (ELISA) kits were used to measure the concentrations of MDA (Beyotime, S0131S), HMGB1 (Shino-Test Corporation, 326070442), and DHA (MyBioSource, MBS2025500) in the indicated samples according to the manufacturers’ instructions.

### Immunofluorescence analysis and H&E staining

Cells were seeded into confocal dishes (Thermo Scientific, 150680) and grown to 30–50% confluency. Cells were washed with PBS and fixed with 4% paraformaldehyde for 30 min, then permeabilized with 0.5% Triton X-100 in PBS for 15 min at room temperature. After blocking with 3% BSA for 30 min, cells were incubated with primary antibody (1:200-1:500) overnight at 4 °C. Subsequently, cells were incubated with fluorescein isothiocyanate (FITC)-conjugated secondary antibody (Immunoway, 1:1000, RS23220) for 1 h, and nuclei were stained with Hoechst for 15 min at room temperature. Finally, images were acquired under a confocal laser scanning microscope (Nikon) and analyzed using NIS-Eliments software. For H&E staining, tissues were fixed with 10% buffered formalin, dehydrated in ethanol, embedded with paraffin, and stained with hematoxylin and eosin as our previously described^77^.

### Molecular docking

The molecular docking modeling was employed to study the interaction of free fatty acids (DHA and DPA) and SLC47A1 (based on PDB ID: 5YCK)^78^. The small molecules—fatty acid was docked to the proteins using online tools (http://www.yinfotek.com/). The fatty acids and the protein structures were converted from pdb into pdbqt format using online tools (http://www.yinfotek.com/). The best model was selected, in which the small molecule was bound at the catalytic site of SLC47A1 with the highest binding affinity (the lowest binding energy).

### Bioinformatics analysis

Three open-access databases, including JASPAR (https://jaspar.genereg.net/), PROMO (http://alggen.lsi.upc.es/cgi-bin/promo_v3/promo/promoinit.cgi?dirDB=TF_8.3), and hTFtarget (http://bioinfo.life.hust.edu.cn/hTFtarget/#!/), were used to predict TF binding sites. The relative profile score threshold was set at 80%. To filter the predictions, in JASPAR, the sequence-matching score was above 10; in PROMO, dissimilarity was under 15%; and in hTFtarget, confidence scores were above 10.

### Statistical analysis

GraphPad Prism (version 8.4.3) was used to collect and analyze data. The statistical analyses used in this study are indicated in the respective figure legends and source data. In general, data with two groups were analyzed by Student unpaired t-test to determine statistically significant effects. Data with multiple groups were analyzed by one-way or two-way ANOVA to determine statistically significant effects. Significance levels are marked using the following footnotes: *p < 0.05, **p < 0.01, ***p < 0.001, and ****p < 0.0001.

### Reporting summary

Further information on research design is available in the Nature Portfolio Reporting Summary linked to this article.

## Supplementary information

Supplementary Information

Description of Additional Supplementary Files

Supplementary Data 1

Supplementary Data 2

Supplementary Data 3

Supplementary Data 4

Supplementary Data 5

Supplementary Data 6

Supplementary Data 7

Reporting Summary

## Data Availability

Original lipidomic profiling data are deposited in MetaboLights database under accession code MTBLS5960. All the data supporting the findings of this study are available within the article and its supplementary information files and from the corresponding author upon reasonable request. Source data are provided with this paper.

## Source data

Source Data
