# Supplementary material for: The lipid flippase SLC47A1 blocks metabolic vulnerability to ferroptosis
Source: Nat Commun. 2022 Dec 27;13:7965. doi: 10.1038/s41467-022-35707-2 (PMC9794750; doi:10.1038/s41467-022-35707-2)
Supplement: Supplementary file 1 — Supplementary Information [file 41467_2022_35707_MOESM1_ESM.pdf]

Supplemental figures and figure legends

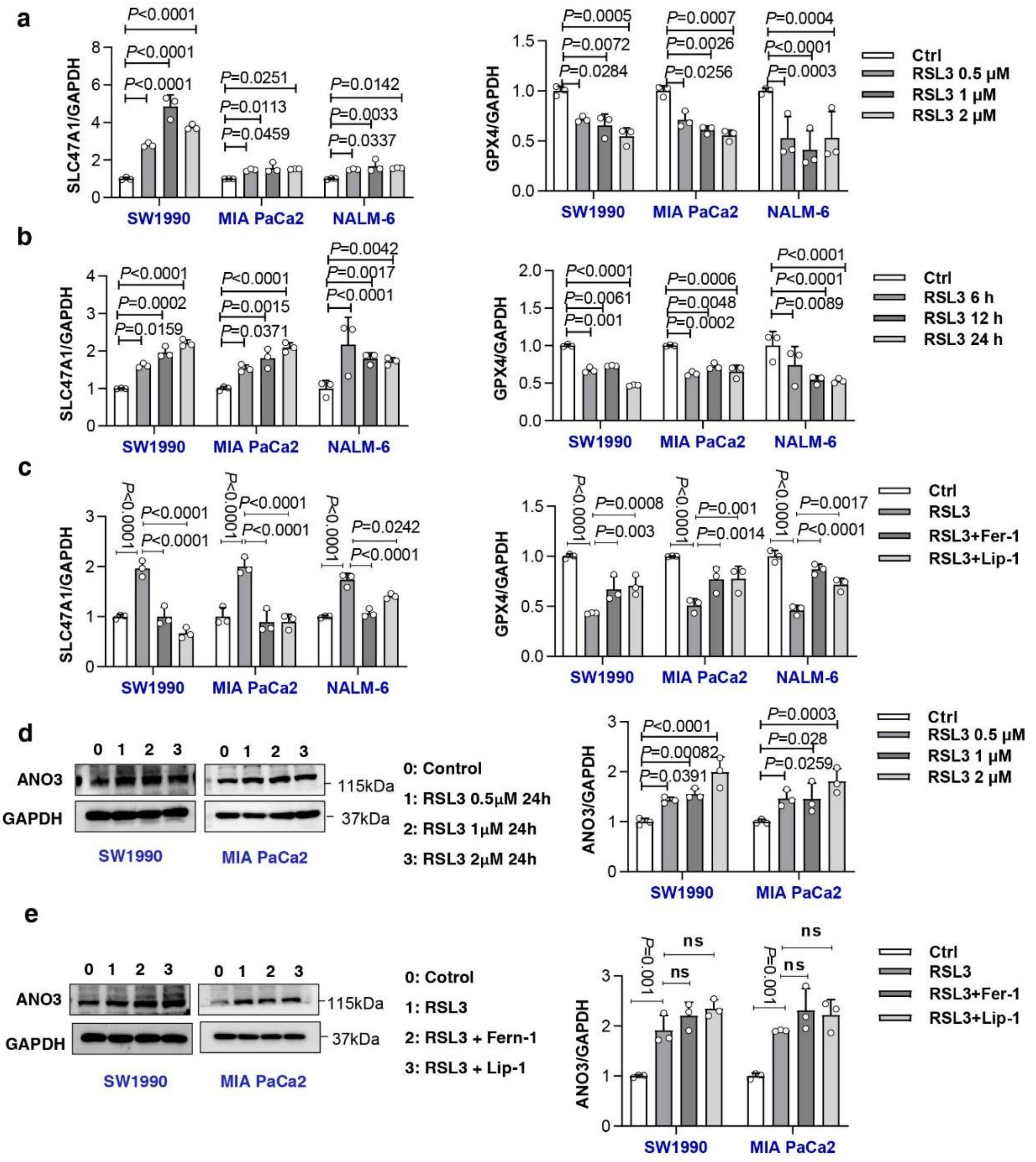

Figure S1. SLC47A1 is upregulated during ferroptosis.

(a-c) Quantification results of immunoblots related to Fig.1b, c and d. **d** Western blot analysis of protein expression in indicated cells following treatment with RSL3 (0.5-2 μM) for 24 h. **e** Western blot analysis of protein expression in indicated cells following treatment with RSL3 (1 μM) for 6-24 h. (**a-e**) Data are shown with 3 biologically independent samples, data were presented as mean±SD; statistical significance was analyzed using one-way ANOVA with Dunnett's multiple comparisons test. Exact p values provided as source data. Source data are provided as a source data file.

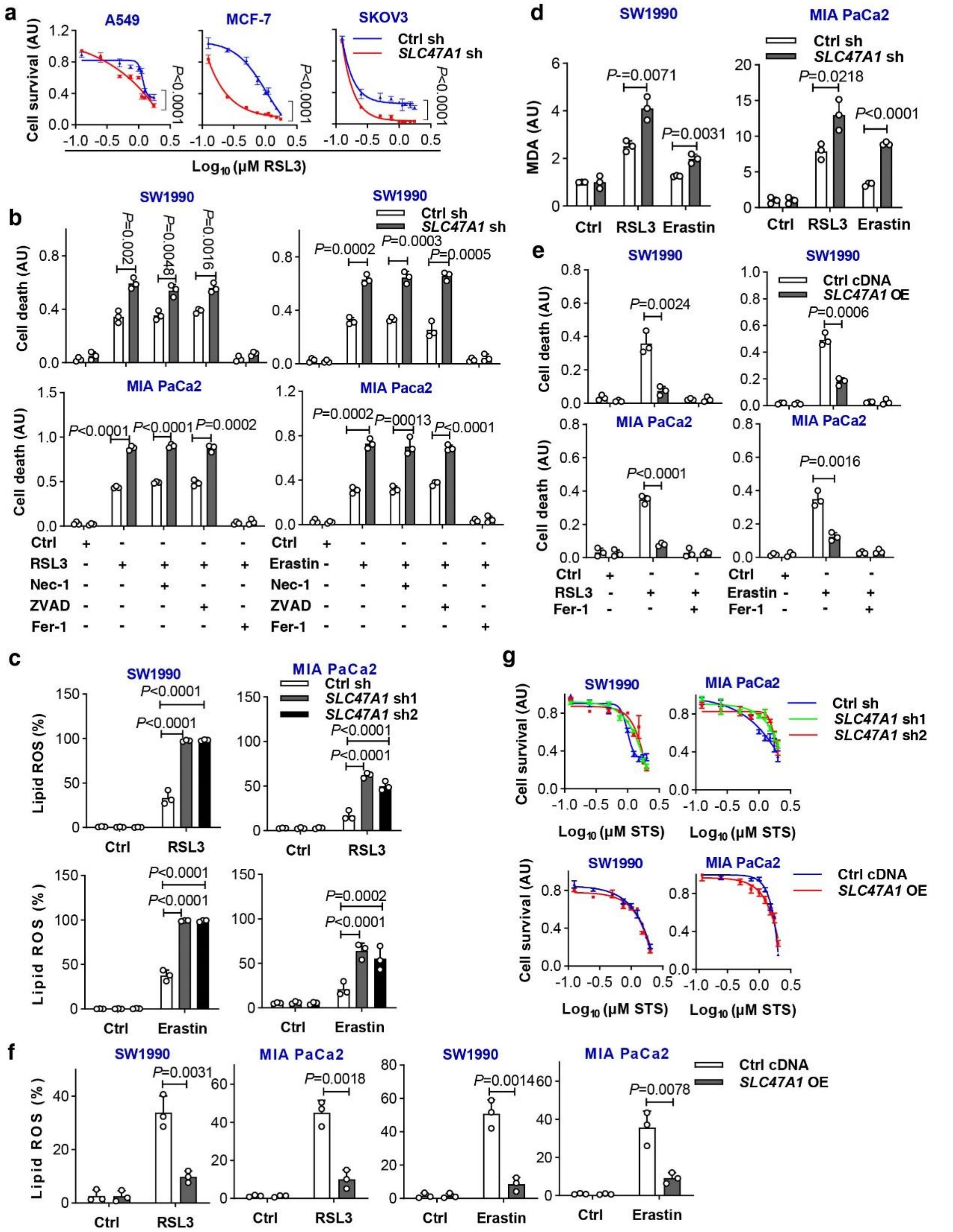

Figure S2. SLC47A1 is a repressor of ferroptosis.

a Cell viability of indicated cells following treatment with RSL3 (1 µM) or erastin (10 µM) for 24 h. **b** Cell death in control and *SLC47A1*-knockdown cells following treatment with RSL3 (1 µM) or erastin (10 µM) in the absence or presence of liproxstatin-1 (Lip-1; 0.5 µM), ferrostatin-1 (Fer-1; 0.5 µM), ZVAD-FMK (20 µM), or necrostatin-1 (Nec-1; 1 µM) for 24 h. **c** Quantification results of lipid ROS correlated to Fig. 2c. **d** MDA of indicated cells following treated with RSL3 (1 µM) or erastin

(10 μM) for 24h. **e** Cell death in control and *SLC47A1*-overexpression cells following treatment with RSL3 (1 μM) or erastin (10 μM) in the absence or presence of ferrostatin-1 (Fer-1; 0.5 μM) for 24 h. **f** Quantification results of lipid ROS related to Fig. 2f. **g** Cell viability of indicated cells following treatment with staurosporine (1 μM) for 12 h. (**a-g**) Data are shown with 3 biologically independent samples, data were presented as mean±SD; statistical significance was analyzed using two-way ANOVA with Dunnett's multiple comparisons test (**a, g**) or unpaired two-tailed T-test (**b, d, e, f**) one-way ANOVA with Dunnett's multiple comparisons test (**c**) . Exact p values provided as source data. Source data are provided as a source data file.

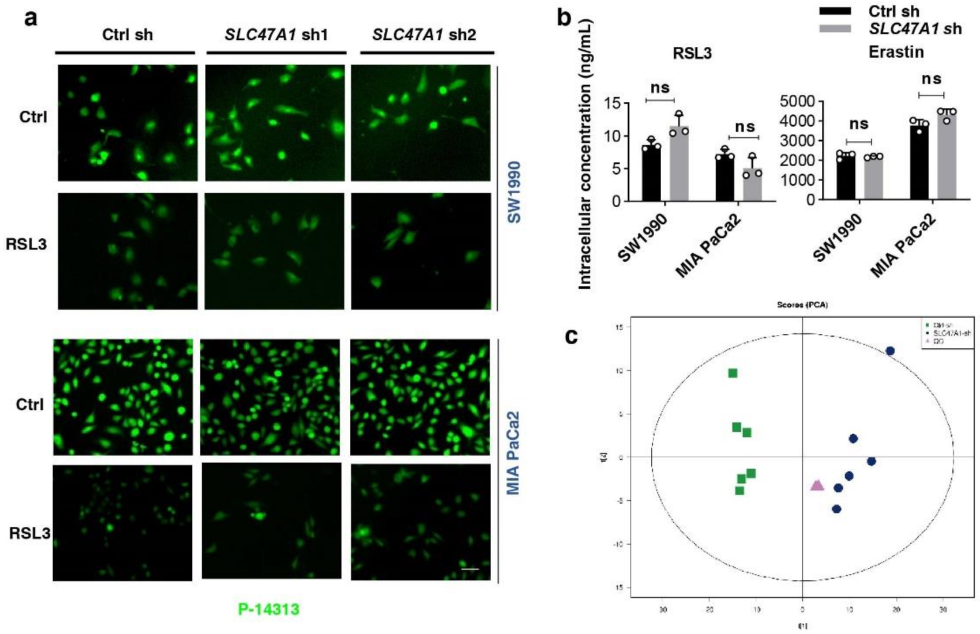

Figure S3. Effects of SLC47A1 on iron accumulation and drug absorption in ferroptosis.

a Analysis of intracellular Fe^{2+} using PG SK staining in indicated cells following treatment with RSL3 (1 μM) for 24 h. The data is representative of three independent experiments.Scale bar=50 μm. **b** LC-MS analysis of intracellular drug levels in indicated cells following treatment with RSL3 (1 μM) and erastin (10 μM) for 24 h (n = 3 biologically independent samples). Data are shown with 3 biologically independent samples, data were presented as mean±SD; statistical significance was analyzed using unpaired two-tailed T-test. ns= not significant. Exact p values provided as source data. Source data are provided as a source data file. **c** Unsupervised Principal Component Analysis (PCA score plot) of extracted lipid features.

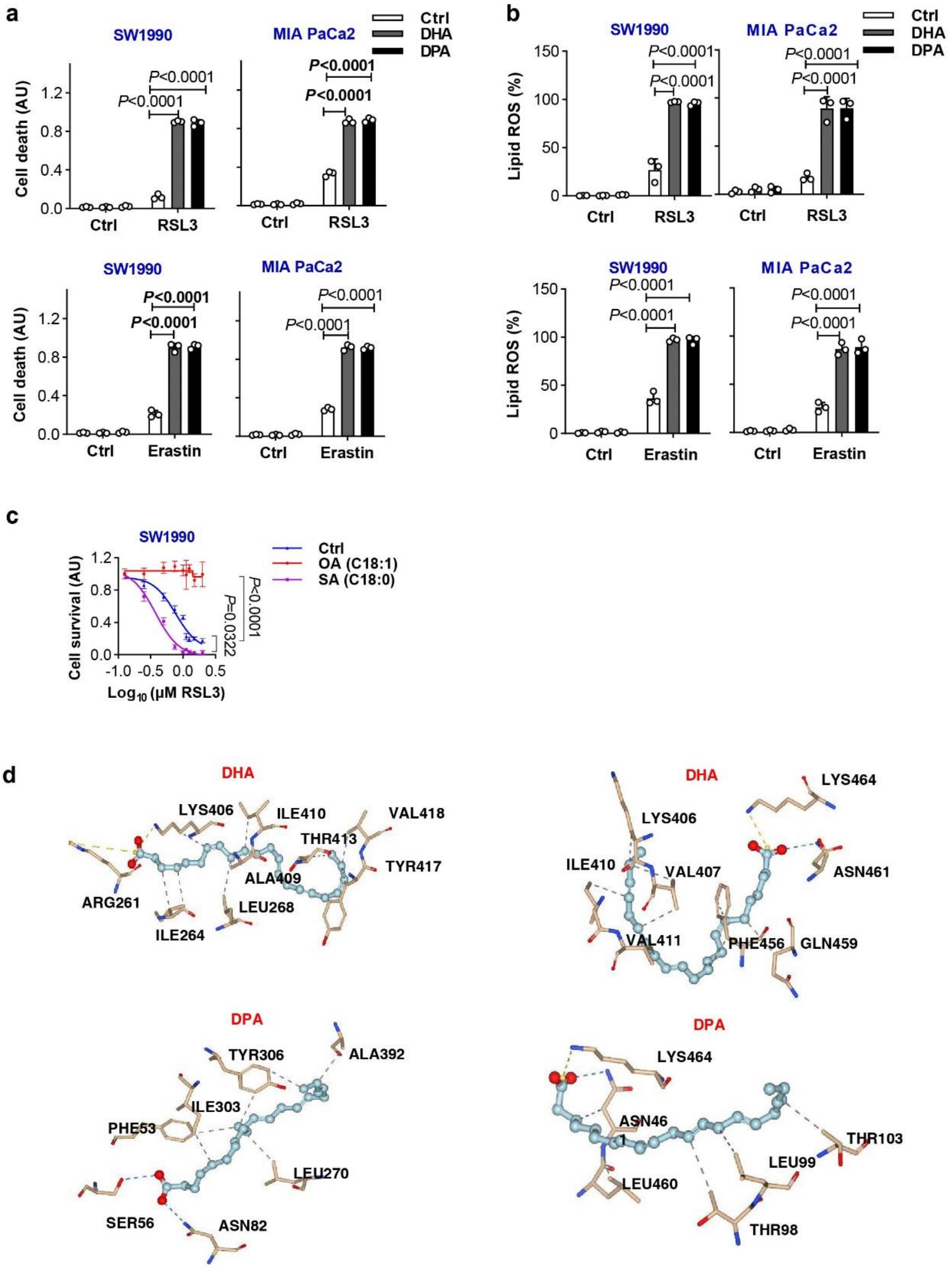

Figure S4. DHA/DPA contributes to the ferroptotic sensitivity in SLC47A1-depletion cells

a Cell death of indicated cells that were pre-treated with DHA (20 μM) and DPA (20 μM) for 3 days, and then treated with RSL3 or erastin for 24 h. **b** Quantification results of lipid ROS related to Fig. 4b. **c** Cell viability of SW1990 which were pre-treated with SA (20 μM) and OA (100 μM) for 3 days, following treatment with RSL3

(1 μM) for 24 h. **d** The binding modes with lowest binding energy and key residues for interaction between SLC47A1 and DHA or DPA. **(a-c)** Data are shown with 3 biologically independent samples, data were presented as mean±SD; statistical significance was analyzed using one-way ANOVA (**a, b**) or two-way ANOVA (**c**) with Dunnett's multiple comparisons test. Exact p values provided as source data. Source data are provided as a source data file.

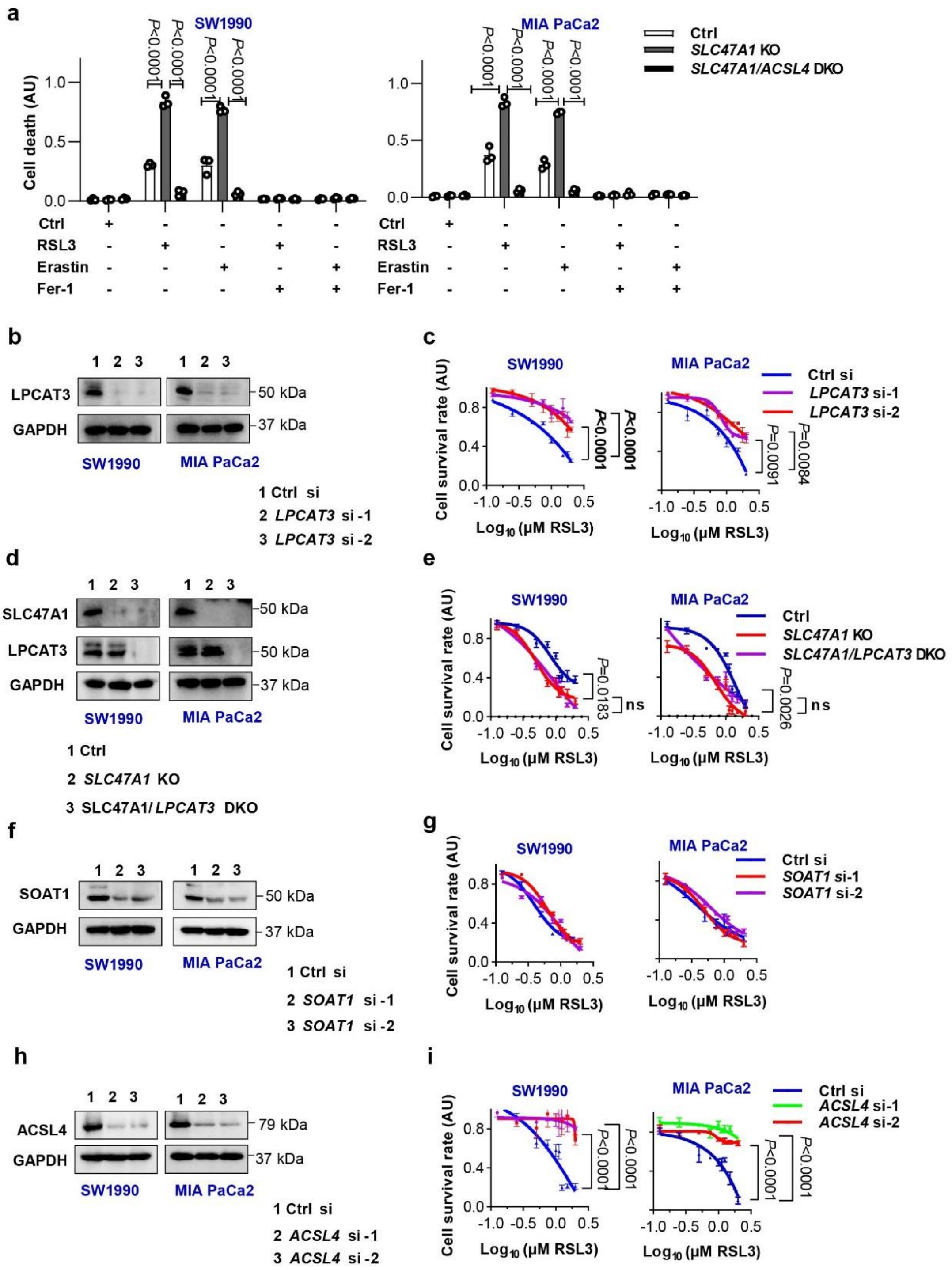

Figure S5. The ACSL4-SOAT1 axis mediates enhanced ferroptosis sensitivity caused by SLC47A1 deficiency.

a Cell death of the indicated cells following treatment with RSL3 (1 μM) or erastin (10 μM) in the absence or presence of ferrostatin-1 (Fer-1; 0.5 μM) for 24 h. **b** Western blot analysis of protein expression in indicated gene knockdown cells. **c** Cell viability of indicated cells following treat with RSL3 for 24 h. **d** Western blot analysis of protein expression in indicated gene knockout cells. DKO: double knockout. **e** Cell

viability of indicated cells following treat with RSL3 for 24 h. **f** Western blot analysis of protein expression in indicated gene knockdown cells. **g** Cell viability of indicated cells following treat with RSL3 for 24 h. **h** Western blot analysis of protein expression in indicated gene knockdown cells. **i** Cell viability of indicated cells following treat with RSL3 for 24 h. (**a, c, e, g, i**) Data are shown with 3 biologically independent samples, data were presented as mean±SD; statistical significance was analyzed using one-way ANOVA (**a**) or two-way ANOVA (**c, e, g, i**) with Dunnett's multiple comparisons test. Exact p values provided as source data. Source data are provided as a source data file. (**b, d, f, h**) The data is representative of three independent experiments.

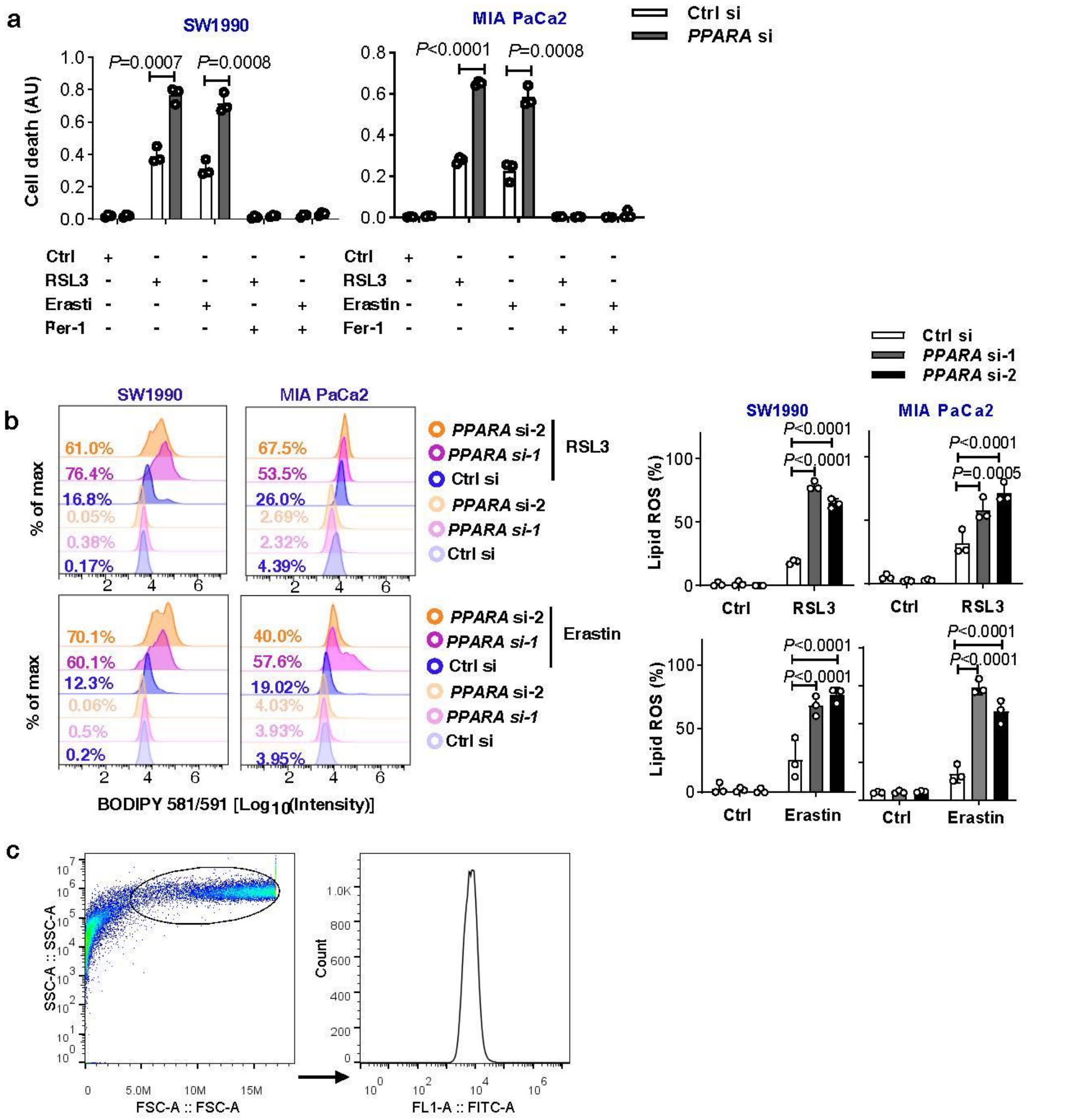

Figure S6. PPARA is required for SLC47A1 upregulation during ferroptosis.
a Cell death of the indicated cells following treatment with RSL3 (1 μM) or erastin (10 μM) in the absence or presence of ferrostatin-1 (Fer-1; 0.5 μM) for 24 h. **b** Indicated cells were treated with RSL3 (1 μM) or erastin (10 μM) for 6 h, and then lipid ROS was measured. The data is representative of three independent experiments. **c** Example gating strategy for flow cytometry analysis of BODIPY-C11 581/591 oxidation in cells. (**a, b**) Data are shown with 3 biologically independent samples, data were presented as mean±SD; statistical significance was analyzed using one-way ANOVA with Dunnett's multiple comparisons test. Exact p values provided as source data. Source data are provided as a source data file.
