# Supplementary material for: The lipid flippase SLC47A1 blocks metabolic vulnerability to ferroptosis
Source: Nat Commun. 2022 Dec 27;13:7965. doi: 10.1038/s41467-022-35707-2 (PMC9794750; doi:10.1038/s41467-022-35707-2)
Supplement: Supplementary file 2 — Description of Additional Supplementary Files [file 41467_2022_35707_MOESM2_ESM.pdf]

Description of Additional Supplementary Files

File name: Supplementary Data 1
Description: The reagent and resource information.

.

File name: Supplementary Data 2
Description: The sequence of a pooled siRNA library targeting phospholipid transporter genes.

File name: Supplementary Data 3
Description: The sequences of sgRNA oligonucleotides and primers.

File name: Supplementary Data 4
Description: The sequence of primers.

File name: Supplementary Data 5
Description: The internal standards for different lipid species identification.

File name: Supplementary Data 6
Description: Median-normalized lipidomic datasets.

File name: Supplementary Data 7
Description: Median-normalized fatty acid datasets.
