# Supplementary material for: The lipid flippase SLC47A1 blocks metabolic vulnerability to ferroptosis
Source: Nat Commun. 2022 Dec 27;13:7965. doi: 10.1038/s41467-022-35707-2 (PMC9794750; doi:10.1038/s41467-022-35707-2)
Supplement: Supplementary file 3 — Supplementary Data 1 [file 41467_2022_35707_MOESM3_ESM.docx]

**KEY RESOURCES TABLE**

| REAGENT or RESOURCE | SOURCE | IDENTIFIER |
| --- | --- | --- |
| **Antibodies** |  |  |
| SLC47A1 (Rabbit polyAb) | Proteintech | Cat# 20898-1-AP, RRID:AB_2878759 |
| SLC47A1 (Rabbit polyAb) (ICC/IF) | Abcam | Cat# ab92295,  RRID: AB_10561617 |
| ANO3 (Rabbit polyAb) | Proteintech | Cat#19489-1-AP, RRID:AB_10638489 |
| GAPDH (Rabbit polyAb) | Affinity | Cat# AF7021, RRID:AB_2839421 |
| GPX4 (Rabbit mAb) | Abcam | Cat# ab125066, RRID:AB_10973901 |
| PPARA (Rabbit polyAb) | Abcam | Cat# ab227074, RRID:N/A |
| ACSL4 (Rabbit mAb) | Abcam | Cat# ab155282, RRID:AB_2714020 |
| SOAT1 (Rabbit polyAb) | Abcam | Cat# ab39327, RRID:AB_778001 |
| LPCAT3(Rabbit polyAb) | Abcam | Cat# ab232958,  RRID:AB_778001 |
| **Chemicals** |  |  |
| RSL3 | Selleck Chemicals | S8155 |
| Erastin | Selleck Chemicals | S7242 |
| Ferrostatin-1 | Selleck Chemicals | S7243 |
| Liproxstatin-1 | Selleck Chemicals | S7699 |
| Z-VAD-FMK | Selleck Chemicals | S7023 |
| Necrostatin-1 | Selleck Chemicals | S8037 |
| Staurosporine | Selleck Chemicals | S1421 |
| DHA | GLPBIO | GC30765 |
| DPA | GLPBIO | GC31637 |
| OA | Selleck Chemicals | S4707 |
| SA | Selleck Chemicals | S5733 |
| WY-14643 | Selleck Chemicals | S8029 |
| GW6471 | Selleck Chemicals | S2798 |
| IKE | Selleck Chemicals | S8877 |
| Cimetidine | Selleck Chemicals | S1845 |
| Puromycin | YEASEN | 60210ES72 |
| Dulbecco’s Modified Eagle’s Medium | Thermo Fisher Scientific | 11995073 |
| Heat-inactivated fetal bovine serum | Thermo Fisher Scientific | A3840001 |
| RPMI-1640 Medium | Thermo Fisher Scientific | 22400097 |
| DMSO | Sigma-Aldrich | 472301 |
| Phosphate buffered saline | Thermo Fisher Scientific | AM9625 |
| Cell lysis buffer | Cell Signaling Technology | 9803 |
| RIPA lysis buffer | Cell Signaling Technology | 9806 |
| 5% nonfat dry milk | Cell Signaling Technology | 9999 |
| Penicillin and streptomycin | Thermo Fisher Scientific | 15070-063 |
| SuperSignal^TM^ West Femto  Maximum Sensitivity Substrate | Thermo Fisher Scientific | 34095 |
| Lipofectamine RNAiMAX | Thermo Fisher Scientific, | 13778-030 |
| PVDF membranes | Millipore | IPVH00010 |
| **Critical commercial assays** |  |  |
| BCA assay kit | Thermo Fisher Scientific | 23225 |
| Cell counting kit-8 kit | YEASEN |  |
| MDA assay kit | Beyotime | S0131S |
| Phen Green™ SK diacetate | Thermo Fisher Scientific | P14313 |
| Hoechst 33342/propidium iodide cell death assay kit | BestBio | BB-4131-1 |
| BODIPY 581/591 C11 probe | Thermo Fisher Scientific | D3861 |
| 10% PAGE Gel Fast Preparation Kit | Epizyme | PG112 |
| 12.5% PAGE Gel Fast Preparation Kit | Epizyme | PG113 |
| RNeasy Plus Micro Kit | QIAGEN | 74034 |
| PrimeScript™ RT Master Mix | Takara | RR036A |
| TB Green® Premix Ex Taq™ II | Takara | RR820Q |
| **Experimental models: cell lines** |  |  |
| MIA PaCa2 | ATCC | CRL-1420 |
| PANC1 | ATCC | CCL-1469 |
| NALM-6 | Fu Heng Biology | FH1161 |
| 293FT | Thermo Fisher Scientific | R70007 |
| A549 | ATCC | CRM-CCL-185 |
| SKOV3 | ATCC | HTB-77 |
| MCF-7 | ATCC | CRL-3435 |
| **Oligonucleotides** |  |  |
| SLC47A1-shRNA1 (sequence: CCGGGACCTATGTCACGATCTTCATCTCGAGATGAAGATCGTGACATAGGTCTTTTTTG) | Beijing Genomics Institution | This paper |
| SLC47A1-shRNA2 (sequence: CCGGCCAGACCTTGTCACGATCTTCTCGAGAAGATCGTGACATAGGTCTGGTTTTTTG) | Beijing Genomics Institution | This paper |
| ACSL4-siRNA1 (sequence: GCAGAUACUCUGGAUAAAUTT) | Gene Pharma | This paper |
| ACSL4-siRNA2 (sequence: CCAAGUAGACCAACGCCUUTT) | Gene Pharma | This paper |
| PPARA siRNA1 (sequence: GCAGGAGGGUAUUGUACAUTT) | Gene Pharma | This paper |
| PPARA siRNA2 (sequence: GGAGCAUUGAACAUC GAAUTT) | Gene Pharma | This paper |
| SOAT1 siRNA1 (sequence: GGACCUGGUGGAUCAUGUUTT) | Gene Pharma | This paper |
| SOAT1 siRNA2 (sequence: GCUCGUGUUCUGGUCCUAUTT) | Gene Pharma | This paper |
| LPCAT3 siRNA1 (sequence: CCUGCUGUGUAUUGUGCUUTT) | Gene Pharma | This paper |
| LPCAT3 siRNA2 (sequence: CCUUCUGGCUGGAUACUAUTT) | Gene Pharma | This paper |
| See Table S2 for sequences of siRNA library | Gene Pharma | Table S2 |
| See Table S4 for primers used  for qPCR | Beijing Genomics Institution | Table S4 |
| **Recombinant DNA** |  |  |
| SLC47A1 cDNA | Genechem | GOSE0286721 |
| **Software and algorithms** |  |  |
| CFX Manager software 2.0 | Bio-Rad | http://www.bio-rad.com/en-us/sku/18 45000-cfx-manager-software?ID=18 45000 |
| GraphPad Prism 8.4.3 | GraphPad | https://www.graphpad.com/scientific-software/prism/ |
| ZEN 2.6 | Zeiss | https://www.zeiss.com.cn/microscopy/pproduct/microscope-software/zen.html #inpagetabs-5 |
| Image Lab ™ Software 3.0 | Bio-Rad | https://www.bio-rad.com/zh-cn/product/ image-lab-software? ID=KRE6P5E8Z |
