# Supplementary material for: The lipid flippase SLC47A1 blocks metabolic vulnerability to ferroptosis
Source: Nat Commun. 2022 Dec 27;13:7965. doi: 10.1038/s41467-022-35707-2 (PMC9794750; doi:10.1038/s41467-022-35707-2)
Supplement: Supplementary file 10 — Reporting Summary [file 41467_2022_35707_MOESM10_ESM.pdf]

nature portfolio

Corresponding author(s): Minghua Yang; Daolin Tang

Last updated by author(s): Dec 7, 2022

Reporting Summary

Nature Portfolio wishes to improve the reproducibility of the work that we publish. This form provides structure for consistency and transparency in reporting. For further information on Nature Portfolio policies, see our Editorial Policies and the Editorial Policy Checklist.

Statistics

For all statistical analyses, confirm that the following items are present in the figure legend, table legend, main text, or Methods section.

n/a	Confirmed	
☐	☒	The exact sample size (*n*) for each experimental group/condition, given as a discrete number and unit of measurement
☐	☒	A statement on whether measurements were taken from distinct samples or whether the same sample was measured repeatedly
☐	☒	The statistical test(s) used AND whether they are one- or two-sided<br>*Only common tests should be described solely by name; describe more complex techniques in the Methods section.*
☒	☐	A description of all covariates tested
☒	☐	A description of any assumptions or corrections, such as tests of normality and adjustment for multiple comparisons
☐	☒	A full description of the statistical parameters including central tendency (e.g. means) or other basic estimates (e.g. regression coefficient) AND variation (e.g. standard deviation) or associated estimates of uncertainty (e.g. confidence intervals)
☐	☒	For null hypothesis testing, the test statistic (e.g. *F*, *t*, *r*) with confidence intervals, effect sizes, degrees of freedom and *P* value noted<br>*Give P values as exact values whenever suitable.*
☒	☐	For Bayesian analysis, information on the choice of priors and Markov chain Monte Carlo settings
☒	☐	For hierarchical and complex designs, identification of the appropriate level for tests and full reporting of outcomes
☒	☐	Estimates of effect sizes (e.g. Cohen's *d*, Pearson's *r*), indicating how they were calculated

Our web collection on statistics for biologists contains articles on many of the points above.

Software and code

Policy information about availability of computer code

Data collection	GraphPad Prism 8.4.3 was used to collect and analyze data.
Data analysis	GraphPad Prism 8.4.3 was used to collect and analyze data. Q-PCR analysis was performed using Bio-Rad CFX Manager software 2.0. Image analysis of lipid peroxidation assay was conducted with using the FlowJo 10 software. Western blots were analyzed using Image Lab Software (Bio-Rad, version 3.0).

For manuscripts utilizing custom algorithms or software that are central to the research but not yet described in published literature, software must be made available to editors and reviewers. We strongly encourage code deposition in a community repository (e.g. GitHub). See the Nature Portfolio guidelines for submitting code & software for further information.

Data

Policy information about availability of data

All manuscripts must include a data availability statement. This statement should provide the following information, where applicable:

- Accession codes, unique identifiers, or web links for publicly available datasets
- A description of any restrictions on data availability
- For clinical datasets or third party data, please ensure that the statement adheres to our policy

All the data supporting the findings of this study are available within the article and its supplementary information files. Original lipidomic profiling data are deposited in MetaboLights database under accession code MTBLS5960. Three open-access databases, including JASPAR (https://jaspar.genereg.net/), PROMO

(http://alggen.lsi.upc.es/cgi-bin/promo_v3/promo/promoinit.cgi?dirDB=TF_8.3), and hTFtarget (http://bioinfo.life.hust.edu.cn/hTFtarget/#!/), were used to predict TF binding sites.

Human research participants

Policy information about studies involving human research participants and Sex and Gender in Research.

Reporting on sex and gender	No human research involved.
Population characteristics	No human research involved.
Recruitment	No human research involved.
Ethics oversight	No human research involved.

Note that full information on the approval of the study protocol must also be provided in the manuscript.

Field-specific reporting

Please select the one below that is the best fit for your research. If you are not sure, read the appropriate sections before making your selection.

☒ Life sciences ☐ Behavioural & social sciences ☐ Ecological, evolutionary & environmental sciences

For a reference copy of the document with all sections, see nature.com/documents/nr-reporting-summary-flat.pdf

Life sciences study design

All studies must disclose on these points even when the disclosure is negative.

Sample size	No statistical methods were used to predetermine sample sizes. Generally accepted sample sizes were used, with reproducible differences between conditions indicating that the chosen sample sizes were sufficient.
Data exclusions	For primary small-molecule sensitivity measurements in multi-well plates individual microtiter wells that exhibit obvious technical errors such as pipetting failure were excluded from analysis.
Replication	Replicates in all assays were confirmatory and the extent described within the text and shown in the main figures.
Randomization	Animal experiments were carried out with randomly chosen littermates of the same sex and matched by age and body weight. For in vitro studies, all cells from multiple dishes were combined and then plated into wells that were treated with various drugs. Thus, all treatment groups came from the same cell stock. Biological independent experiments were performed on independent aliquots of cells thawed from the liquid nitrogen freezer.
Blinding	The investigator and personnel were not blinded during this study in order to allow the investigators to a correct identification of samples and to ensure a correct data collection.

Reporting for specific materials, systems and methods

We require information from authors about some types of materials, experimental systems and methods used in many studies. Here, indicate whether each material, system or method listed is relevant to your study. If you are not sure if a list item applies to your research, read the appropriate section before selecting a response.

Materials & experimental systems

n/a	Involved in the study	
☐	☒	Antibodies
☐	☒	Eukaryotic cell lines
☒	☐	Palaeontology and archaeology
☐	☒	Animals and other organisms
☒	☐	Clinical data
☒	☐	Dual use research of concern

Methods

n/a	Involved in the study	
☒	☐	ChIP-seq
☐	☒	Flow cytometry
☒	☐	MRI-based neuroimaging

Antibodies

Antibodies used	SLC47A1 (Rabbit polyAb) Proteintech Cat# 20898-1-AP,<br>SLC47A1 (Rabbit polyAb) (ICC/IF) Abcam Cat# ab92295,<br>ANO3 (Rabbit polyAb) Proteintech Cat#19489-1-AP,<br>GAPDH (Rabbit polyAb) Affinity Cat# AF7021,<br>GPX4 (Rabbit mAb) [EPNCIR144] Abcam Cat# ab125066,<br>PPARA (Rabbit polyAb) Abcam Cat# ab227074,<br>ACSL4 (Rabbit mAb) [EPR8640] Abcam Cat# ab155282,<br>SOAT1 (Rabbit polyAb) Abcam Cat# ab39327,<br>LPCAT3 (Rabbit polyAb) Abcam Cat# ab232958,<br>Rabbit anti-goat IgG secondary antibody Abcam, Cat# ab6741<br>Fluorescein isothiocyanate (FITC)-conjugated secondary antibody (Rabbit anti-goat) Immunoway, Cat# RS23220
Validation	Below are validation statements from manufactures:<br>SLC47A1 (Rabbit polyAb) Proteintech Cat# 20898-1-AP, WB, https://www.ptgcn.com/products/SLC47A1-Antibody-20898-1-AP<br>SLC47A1 (Rabbit polyAb) (ICC/IF) Abcam Cat# ab92295, ICC/IF, https://www.abcam.cn/mate-1-antibody-ab92295<br>ANO3 (Rabbit polyAb) Proteintech Cat#19489-1-AP, WB, https://www.ptgcn.com/products/ANO3-Antibody-19489-1-AP<br>GAPDH (Rabbit polyAb) Affinity Cat# AF7021, WB, https://www.affbiotech.com/goods-6289-AF7021-GAPDH_Antibody<br>GPX4 (Rabbit mAb) [EPNCIR144] Abcam Cat# ab125066, WB, https://www.abcam.cn/glutathione-peroxidase-4-antibody-epncir144-ab125066<br>PPARA (Rabbit polyAb) Abcam Cat# ab227074, WB, https://www.abcam.cn/ppar-alpha-antibody-chip-grade-ab227074<br>ACSL4 (Rabbit mAb) [EPR8640] Abcam Cat# ab155282, WB, https://www.abcam.cn/facl4-antibody-epr8640-ab155282<br>SOAT1 (Rabbit polyAb) Abcam Cat# ab39327, WB, https://www.abcam.cn/soat-1acat1-antibody-ab39327<br>LPCAT3 (Rabbit polyAb) Abcam Cat# ab232958, WB, https://www.abcam.cn/lpcat3-antibody-ab232958<br>Rabbit anti-goat IgG secondary antibody Abcam, Cat# ab6741, WB, https://www.abcam.cn/rabbit-goat-igg-hl-hrp-ab6741<br>Rluorescein isothiocyanate (FITC)-conjugated secondary antibody (Rabbit anti-goat) Immunoway, Cat# RS23220 ICC/IF, http://www.immunoway.com/Home/Search?keywords=RS23220

Eukaryotic cell lines

Policy information about cell lines and Sex and Gender in Research

Cell line source(s)	SW1990 (CRL-2172), MIA PaCa2 (CRL-1420), HT-1080 (CCL-121), MCF-7(HTB-22), SKOV3 (HTB-77), and A549 (CCL-185) cell lines were obtained from the American Type Culture Collection. An NALM-6 (FH1161) cell line was obtained from Fu Heng Biology (A commercial cell bank from China). 293FT (R70007) cell line was obtained from Thermo Fisher Scientific
Authentication	SW1990, MIA PaCa2, HT-1080, MCF-7, SKOV3, A549,NALM-6 and 293FT cell lines were authenticated by STR profiling.
Mycoplasma contamination	All cell lines used were confirmed to be mycoplasma negative.
Commonly misidentified lines (See ICLAC register)	None of the cell lines used are commonly misidentified lines.

Animals and other research organisms

Policy information about studies involving animals; ARRIVE guidelines recommended for reporting animal research, and Sex and Gender in Research

Laboratory animals	Xenograft studies: 6-to 8-week-old athymic nude female mice (Charles River 490)
Wild animals	No wild animals were used in the study.
Reporting on sex	female mice
Field-collected samples	No field-collected samples were used in the study.
Ethics oversight	We conducted all animal care and experiments in accordance with the Assoclation for Assessment and Accreditation of Laboratory Animal Care guidelines and with approval from our institutional animal care and use committees (Central South University).

Note that full information on the approval of the study protocol must also be provided in the manuscript.

Flow Cytometry

Plots

Confirm that:

☒ The axis labels state the marker and fluorochrome used (e.g. CD4-FITC).

☒ The axis scales are clearly visible. Include numbers along axes only for bottom left plot of group (a 'group' is an analysis of identical markers).

☒ All plots are contour plots with outliers or pseudocolor plots.

☒ A numerical value for number of cells or percentage (with statistics) is provided.

Methodology

Sample preparation	BODIPY 581/591 C11 probe (Thermo Fisher Scientific, D3861) was used to detect lipid R0S according to the manufacturer instructions. In brief, cells were incubated with BODIPY 581/591 C11 at a final concentration of 5 uM for 30 min at 37? and washed three times with PBS. Oxidation of the polyunsaturated butadienyl portion of the dye resulted in a shift of the fluorescence emission peak from 590 nm to 510 nm, which was measured using a BD Accuri C6 Plus flow cytometer (BD Biosciences). A minimum of 10,000 cells were analyzed for each condition.
Instrument	BD Accuri C6 Plus flow cytometer.
Software	BD Accuri software for data collection and FlowJo v10 for data analysis
Cell population abundance	A minimum of 10,000 post-staining cells were analyzed for each condition. BODIPY-493/503 and B0DIPY-C11 signal wasconfirmed on a fluorescent microscope.
Gating strategy	Analyzed for BODIPY-581/591 signal using the FITC 488nM filter, or for BODIPY-C11 using both the FITE and PE-TexasRedfillet

☒ Tick this box to confirm that a figure exemplifying the gating strategy is provided in the Supplementary Information.
