# Supplementary material for: The lipid flippase SLC47A1 blocks metabolic vulnerability to ferroptosis
Source: Nat Commun. 2022 Dec 27;13:7965. doi: 10.1038/s41467-022-35707-2 (PMC9794750; doi:10.1038/s41467-022-35707-2)

Source data-uncropped blots and gels

Fig. 1b

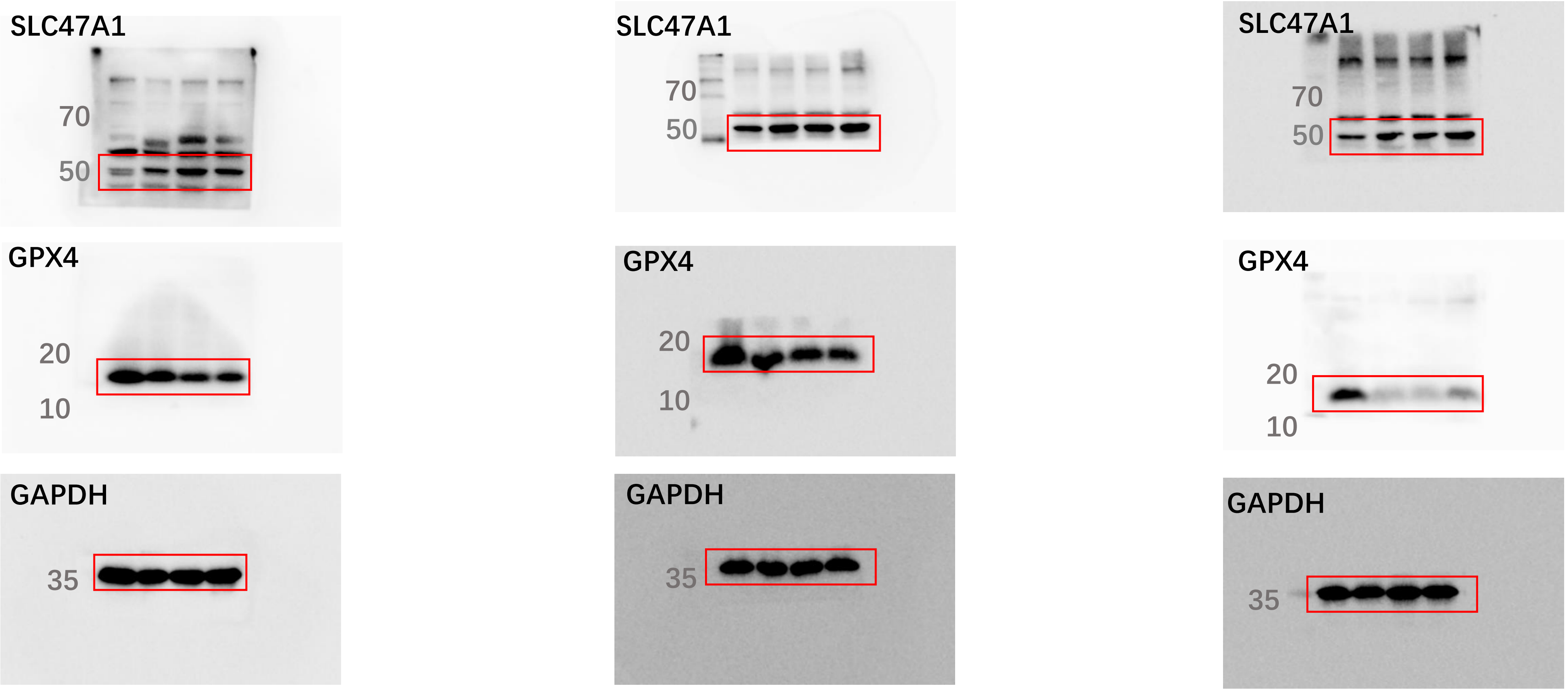
SLC47A1
70
50
GPX4
20
10
GAPDH
35
SLC47A1
70
50
GPX4
20
10
GAPDH
35
SLC47A1
70
50
GPX4
20
10
GAPDH
35

Fig. 1c

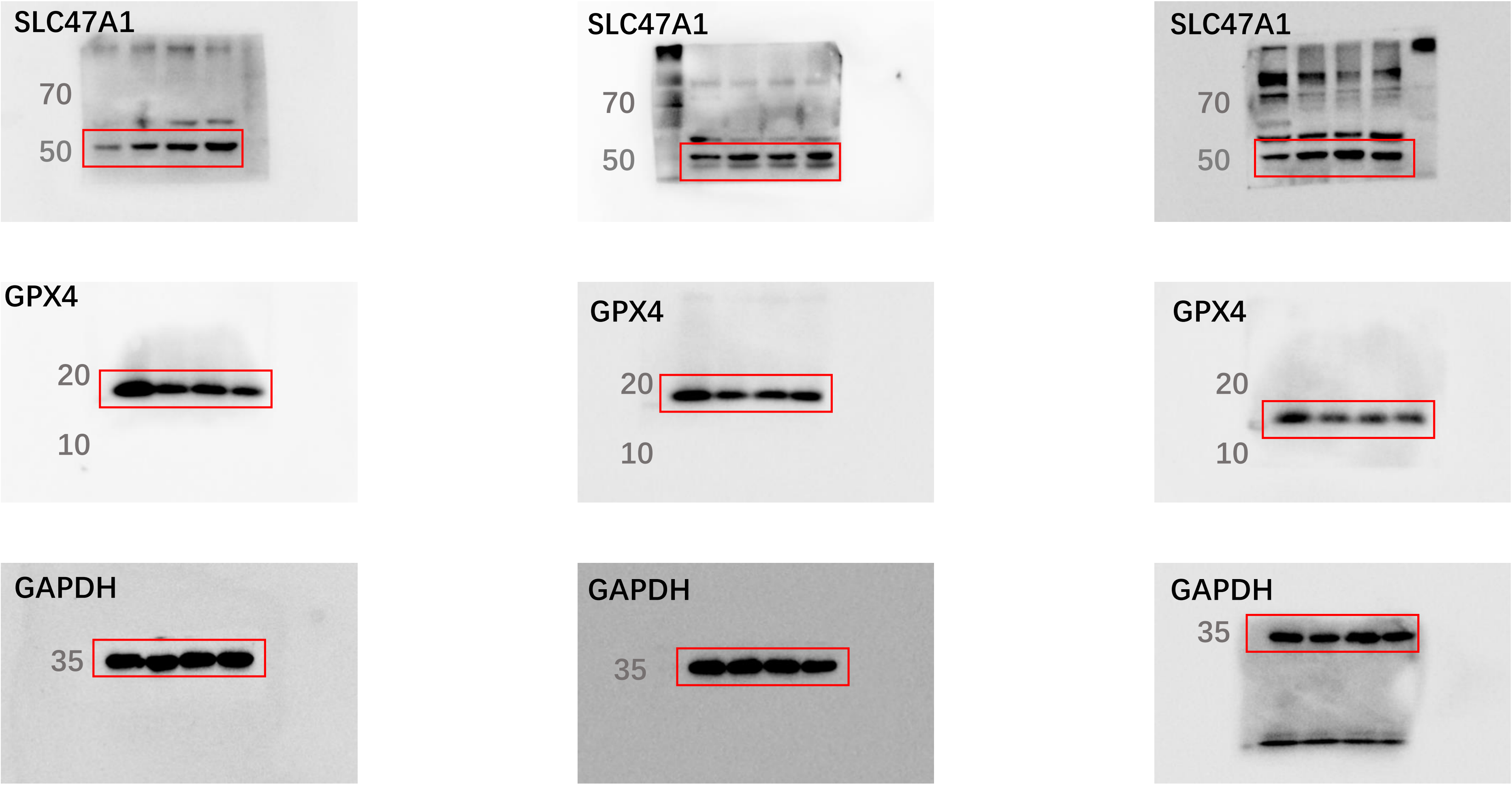
SLC47A1
70
50
SLC47A1
70
50
SLC47A1
70
50
GPX4
20
10
GPX4
20
10
GPX4
20
10
GAPDH
35
GAPDH
35
GAPDH
35

Fig. 1d

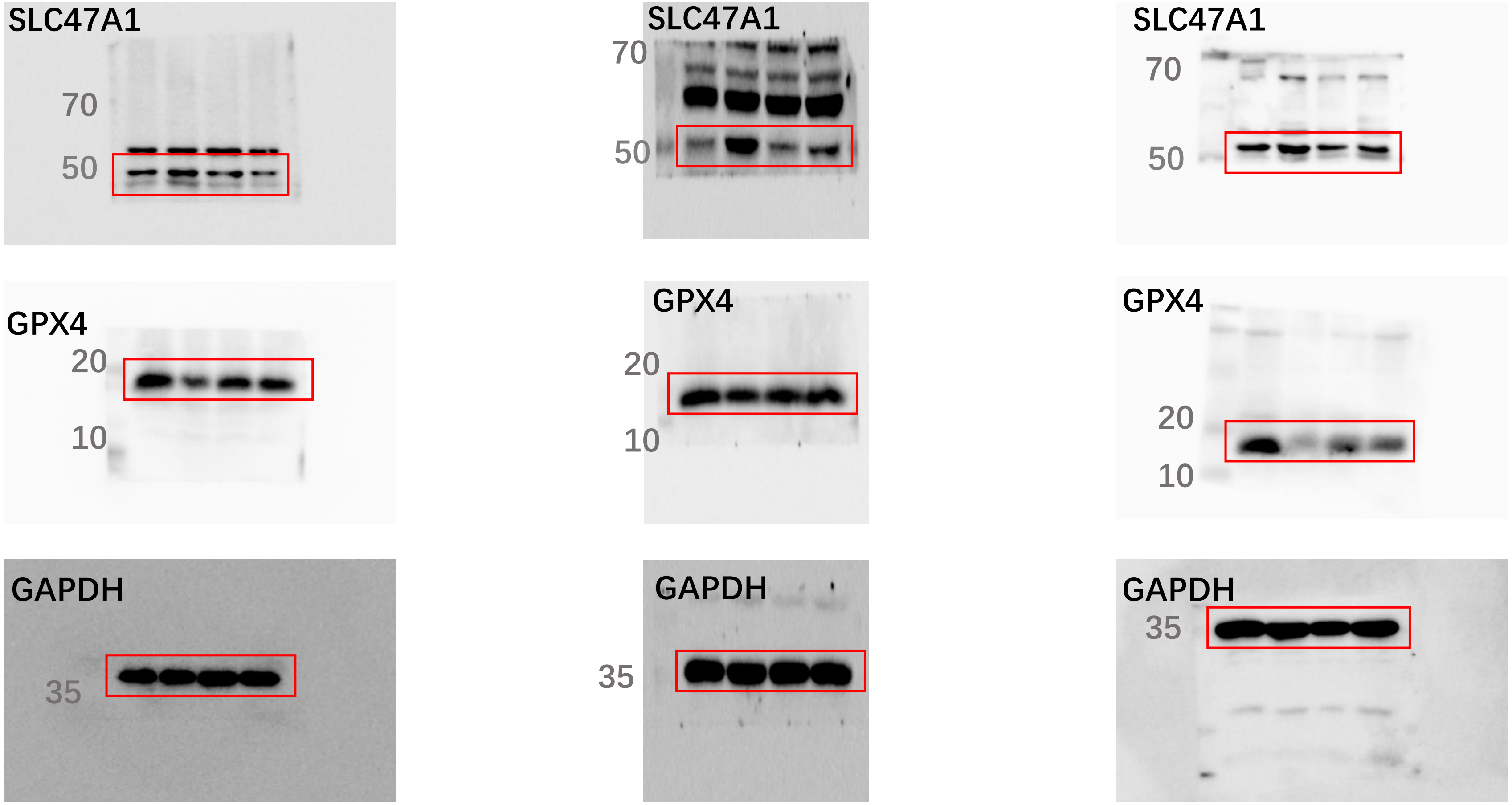
SLC47A1
70
50
SLC47A1
70
50
SLC47A1
70
50
GPX4
20
10
GPX4
20
10
GPX4
20
10
GAPDH
35
GAPDH
35
GAPDH
35

Fig. 1f

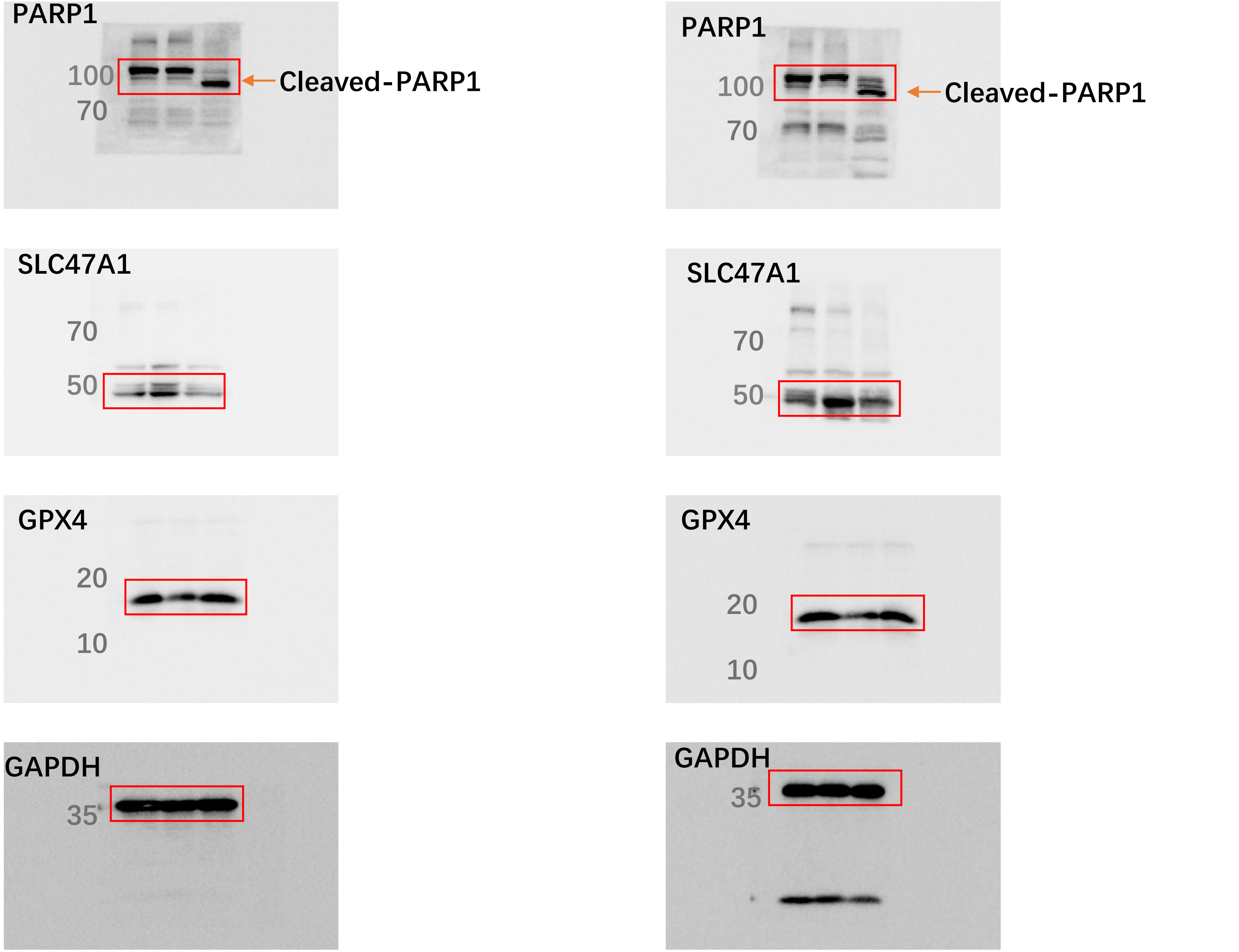
PARP1
100
70
Cleaved-PARP1
SLC47A1
70
50
GPX4
20
10
GAPDH
35
PARP1
100
70
Cleaved-PARP1
SLC47A1
70
50
GPX4
20
10
GAPDH
35

Fig. 2a

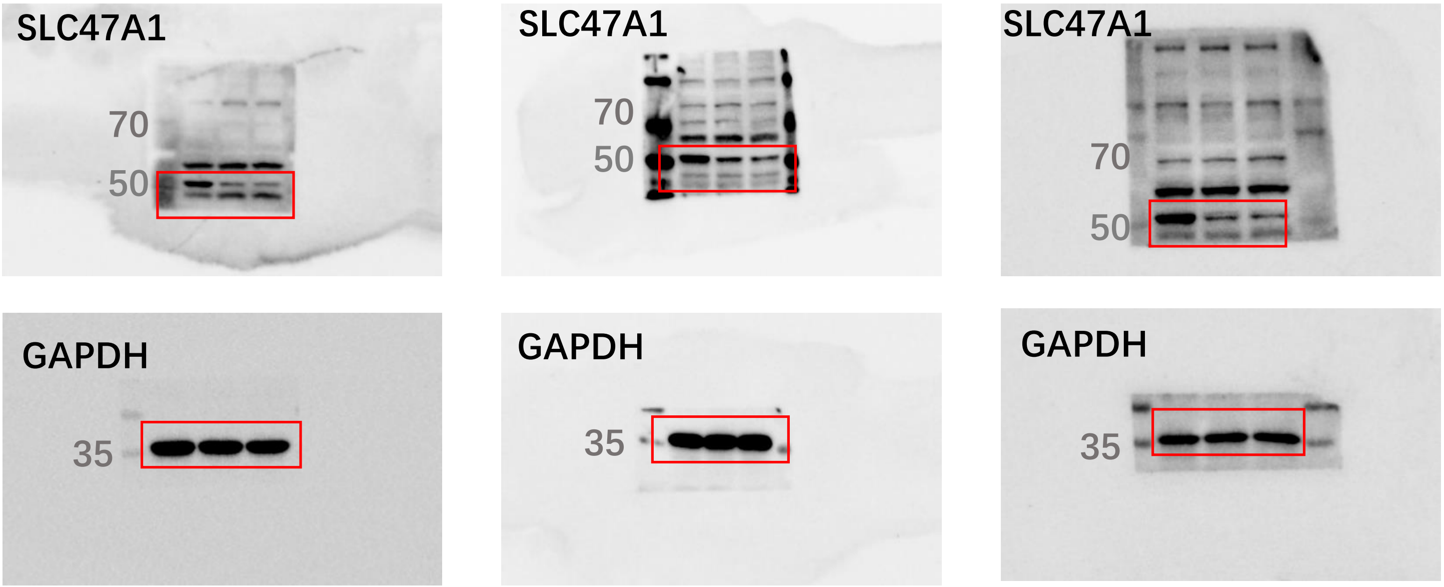
SLC47A1
70
50
SLC47A1
70
50
SLC47A1
70
50
GAPDH
35
GAPDH
35
GAPDH
35

Fig. 2d

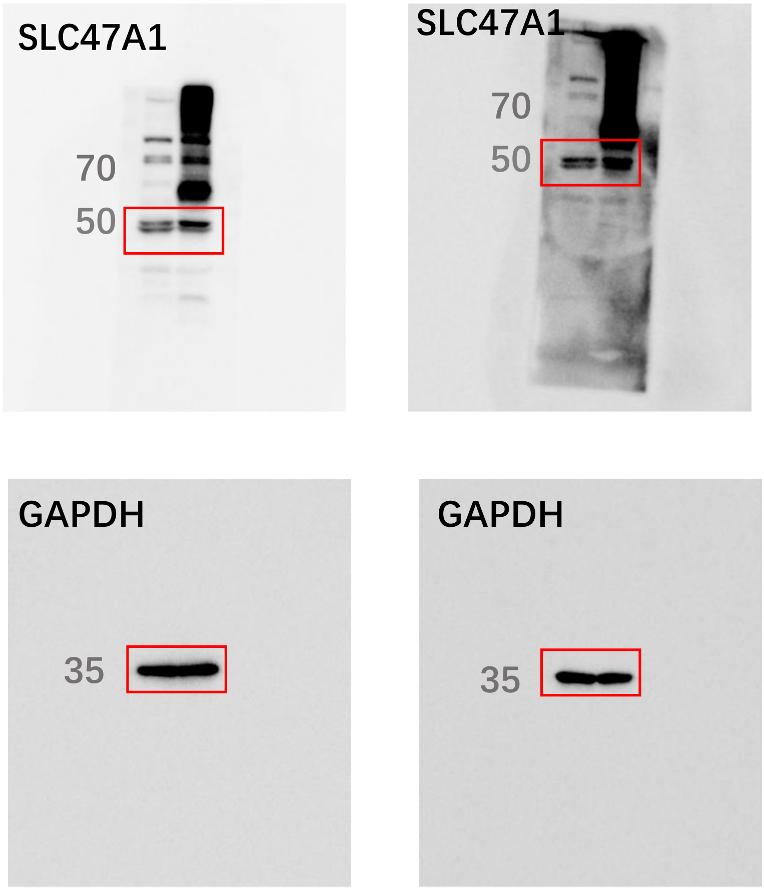
SLC47A1
70
50
SLC47A1
70
50
GAPDH
35
GAPDH
35

Fig. 3a

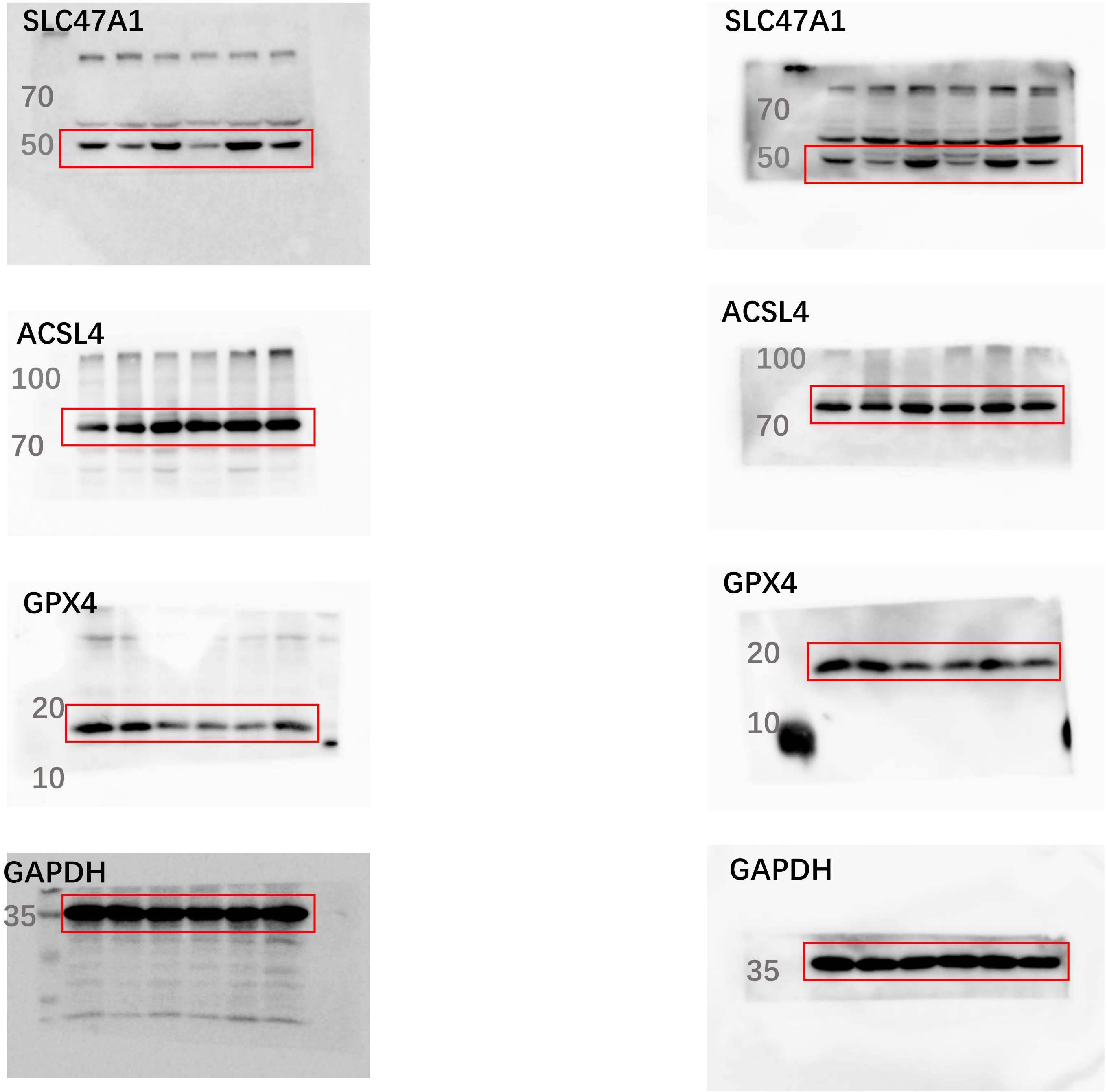
SLC47A1
70
50
ACSL4
100
70
GPX4
20
10
GAPDH
35
SLC47A1
70
50
ACSL4
100
70
GPX4
20
10
GAPDH
35

Fig. 5a

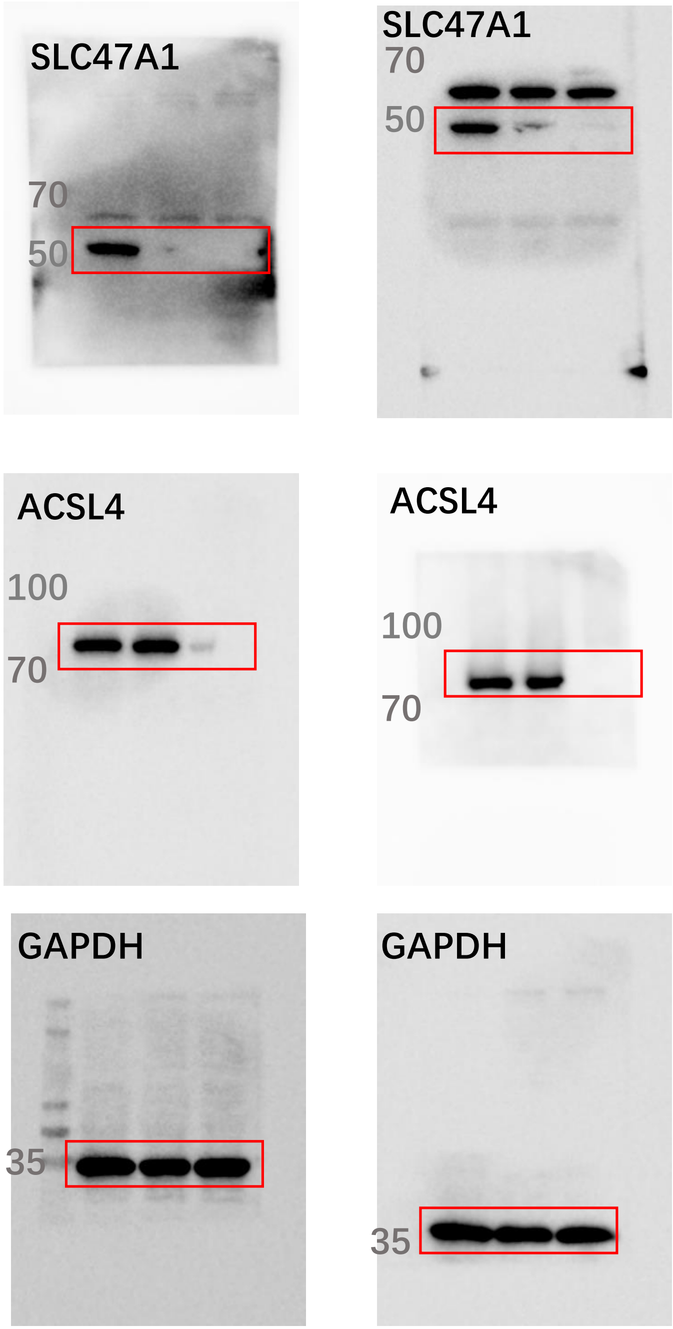
SLC47A1
70
50
SLC47A1
70
50
ACSL4
100
70
ACSL4
100
70
GAPDH
35
GAPDH
35

Fig. 5d

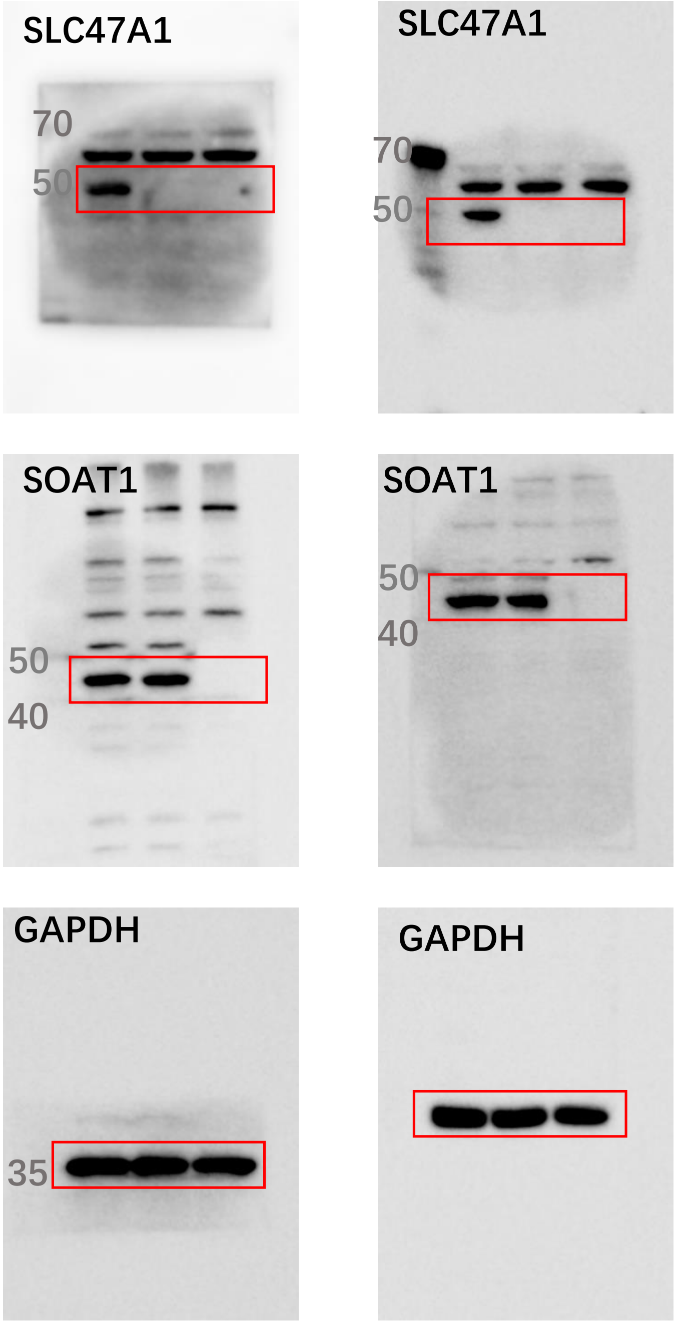
SLC47A1
70
50
SLC47A1
70
50
SOAT1
50
40
SOAT1
50
40
GAPDH
35
GAPDH

Fig. 6c

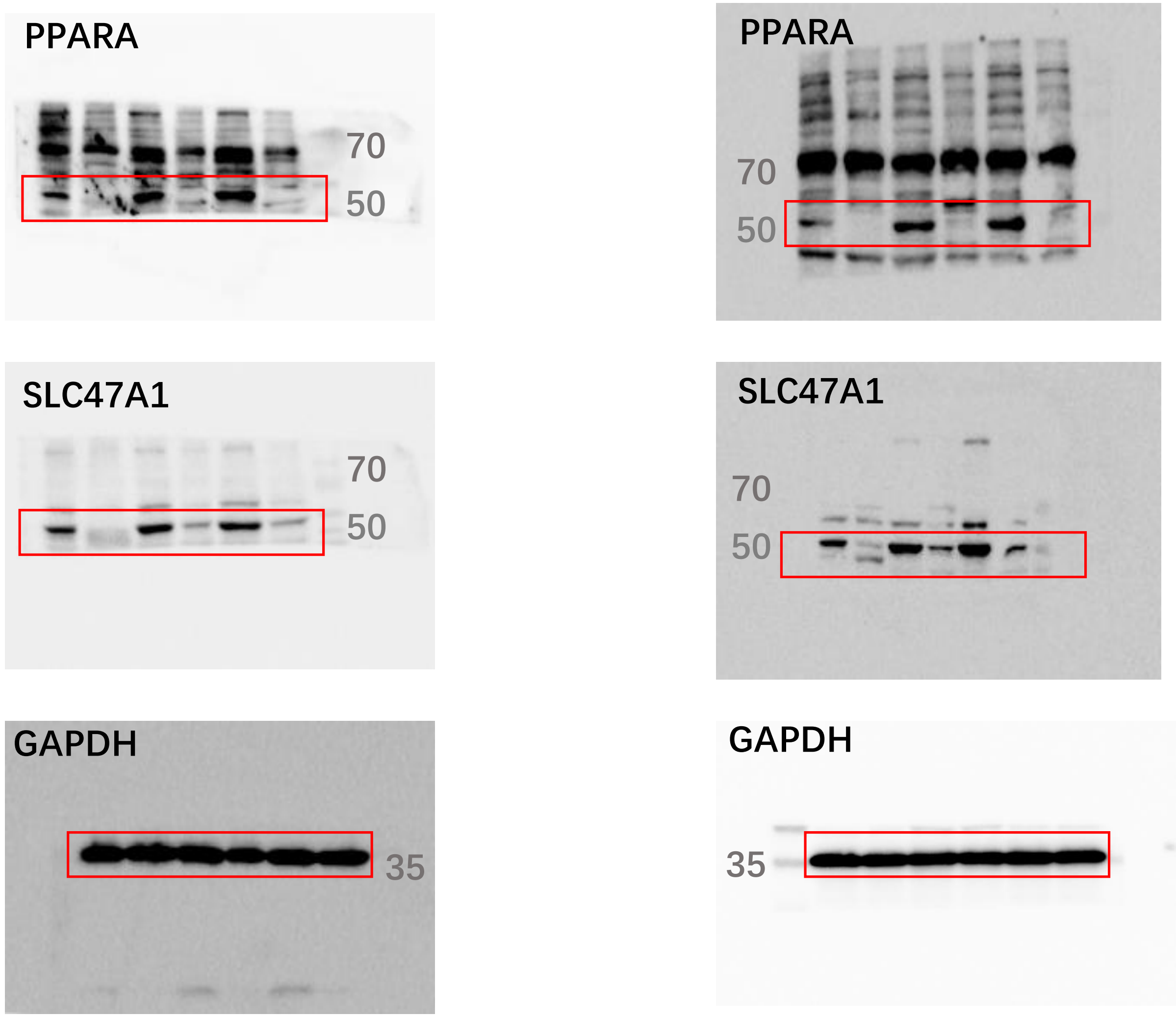

PPARA
70
50
PPARA
70
50
SLC47A1
70
50
SLC47A1
70
50
GAPDH
35
GAPDH
35

Fig. 6d

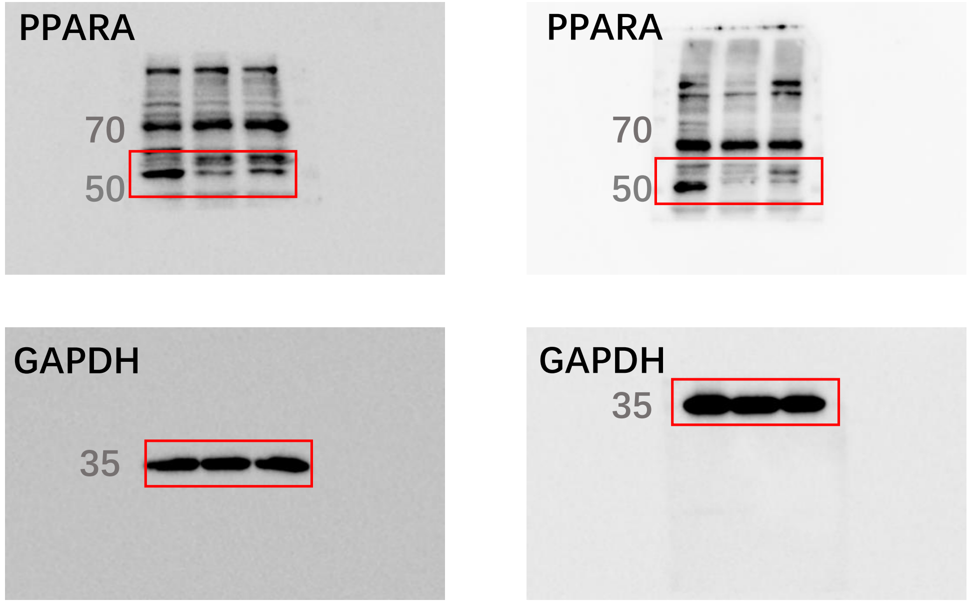
PPARA
70
50
PPARA
70
50
GAPDH
35
GAPDH
35

Fig. 6g

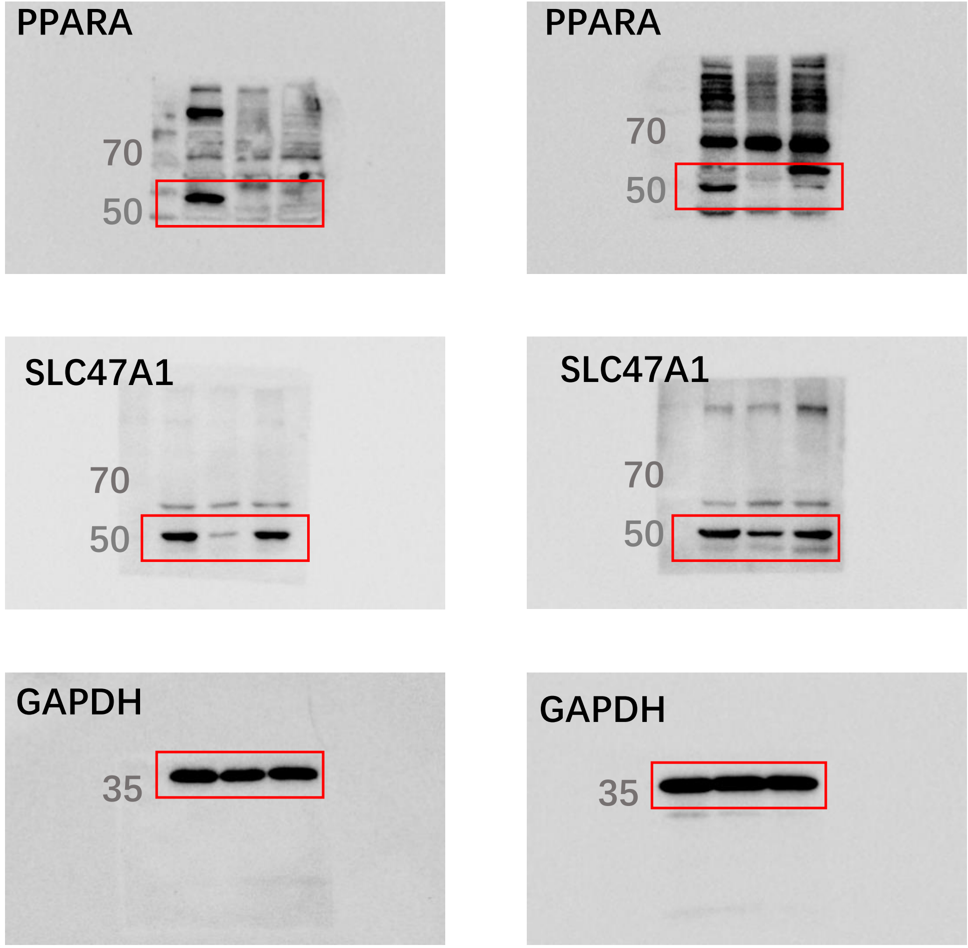
PPARA
70
50
PPARA
70
50
SLC47A1
70
50
SLC47A1
70
50
GAPDH
35
GAPDH
35

Fig. S1

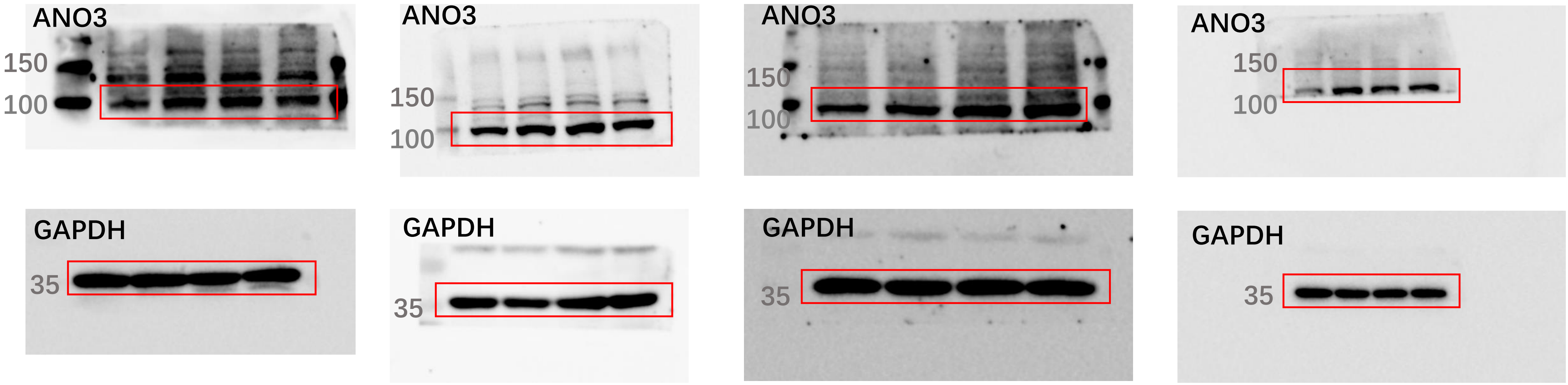
ANO3
150
100
ANO3
150
100
ANO3
150
100
ANO3
150
100
GAPDH
35
GAPDH
35
GAPDH
35
GAPDH
35

Fig. S5b

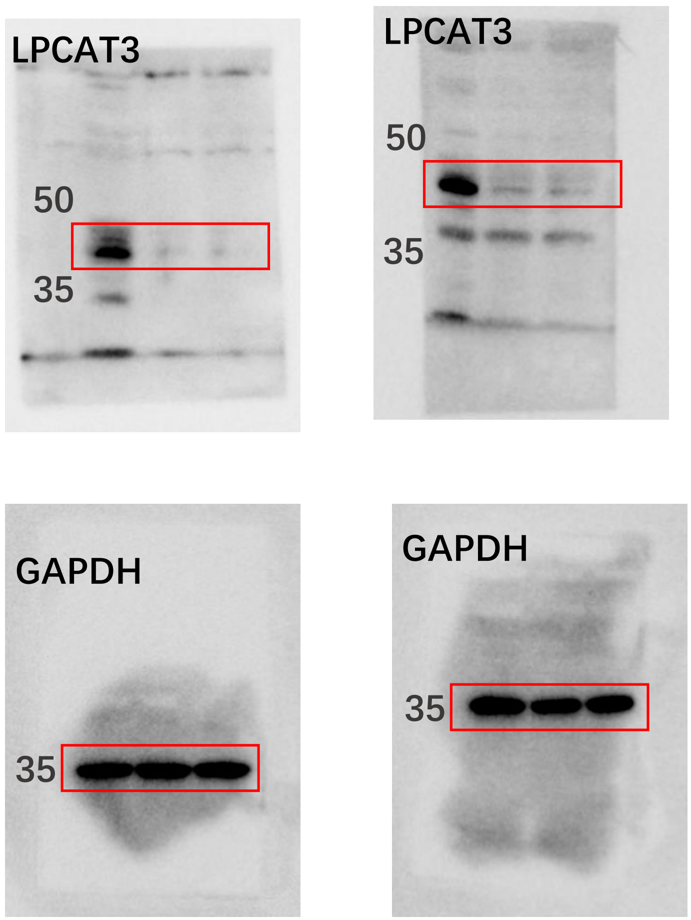

Fig. S5d

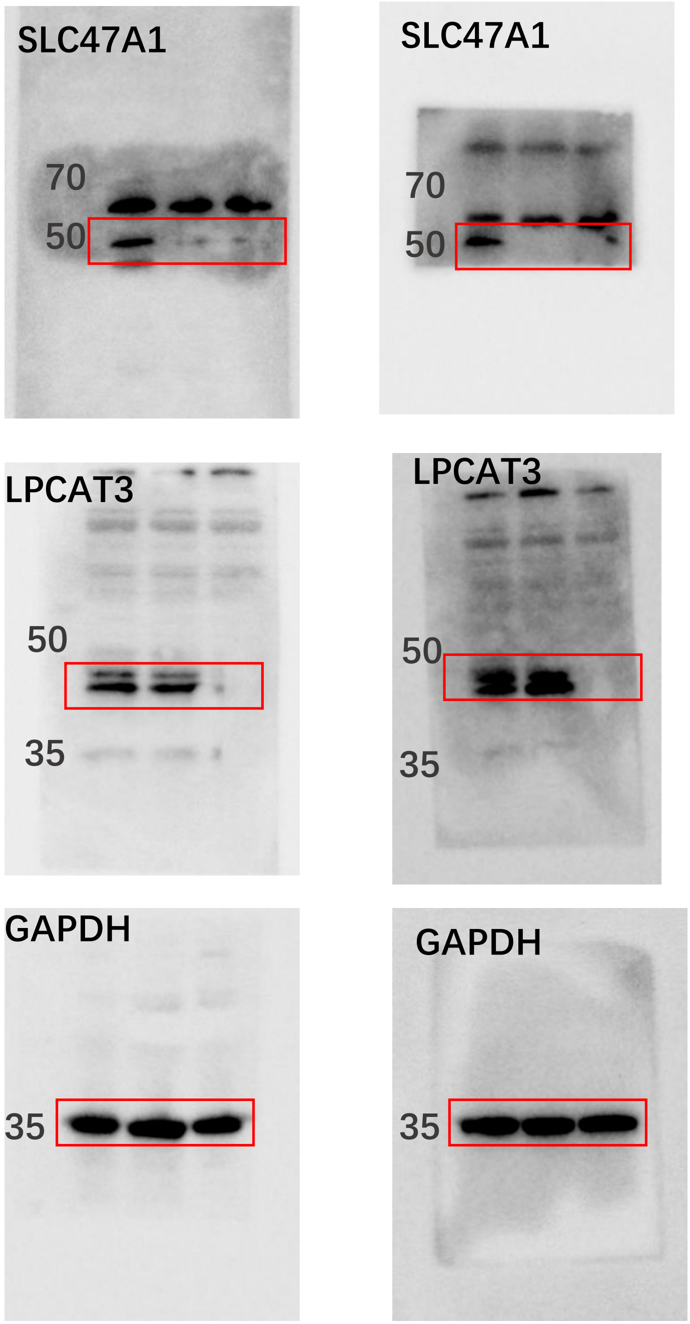

Fig. S5f

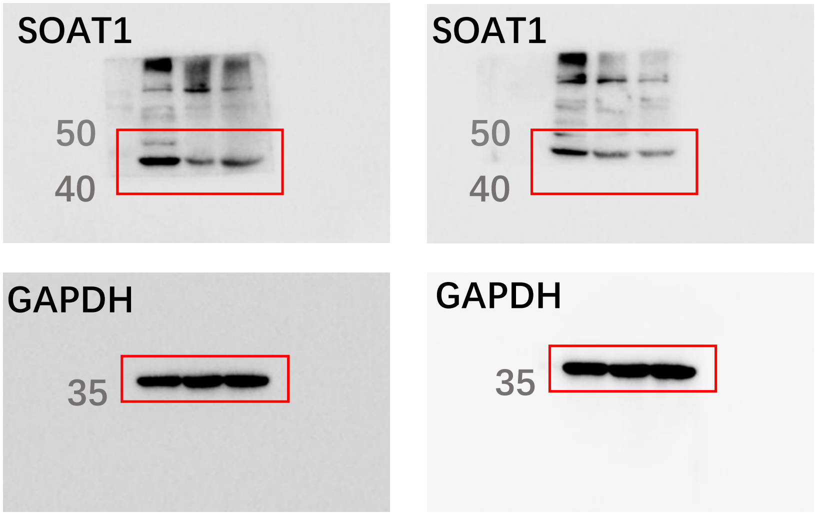

Fig. S5h

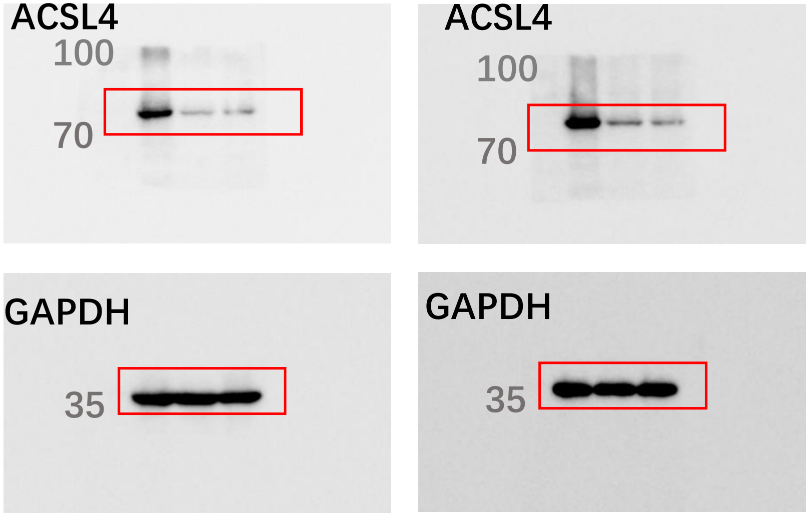

Supplement: Supplementary file 11 — Source Data [file 41467_2022_35707_MOESM11_ESM.zip › Source Data gels.pdf]
